# The United Kingdom Childhood Cancer Study: objectives, materials and methods

**DOI:** 10.1054/bjoc.1999.1045

**Published:** 2000-02-01

**Authors:** UK Childhood Cancer Study Investigators

**Keywords:** childhood cancer, aetiology, radon, magnetic fields, leukaemia classification

## Abstract

An investigation into the possible causes of childhood cancer has been carried out throughout England, Scotland and Wales over the period 1991–1998. All children known to be suffering from one or other type of the disease over periods of 4–5 years have been included, and control children matched for sex, age and area of residence have been selected at random from population registers. Information about both groups of children (with and without cancer) has been obtained from parental questionnaires, general practitioners' and hospital records, and from measurement of the extent of exposure to radon gas, terrestrial gamma radiation, and electric and magnetic fields. Samples of blood have also been obtained from the affected children and their parents and stored. Altogether 3838 children with cancer, including 1736 with leukaemia, and 7629 unaffected children have been studied. Detailed accounts are given of the nature of the information obtained in sections describing the general methodology of the study, the measurement of exposure to ionizing and non-ionizing radiation, the classification of solid tumours and leukaemias, and the biological material available for genetic analysis. © 2000 Cancer Research Campaign


*British Journal of Cancer* (2000) **82**(5), 1073–1102
© 2000 Cancer Research Campaign
DOI: 10.1054/ bjoc.1999.1045, available online at http://www.idealibrary.com on IDEAL®

### The United Kingdom Childhood Cancer Study: objectives, materials and methods

**UK Childhood Cancer Study Investigators***

**Summary** An investigation into the possible causes of childhood cancer has been carried out throughout England, Scotland and Wales over the period 1991–1998. All children known to be suffering from one or other type of the disease over periods of 4–5 years have been included, and control children matched for sex, age and area of residence have been selected at random from population registers. Information about both groups of children (with and without cancer) has been obtained from parental questionnaires, general practitioners' and hospital records, and from measurement of the extent of exposure to radon gas, terrestrial gamma radiation, and electric and magnetic fields. Samples of blood have also been obtained from the affected children and their parents and stored. Altogether 3838 children with cancer, including 1736 with leukaemia, and 7629 unaffected children have been studied. Detailed accounts are given of the nature of the information obtained in sections describing the general methodology of the study, the measurement of exposure to ionizing and non-ionizing radiation, the classification of solid tumours and leukaemias, and the biological material available for genetic analysis. © 2000 Cancer Research Campaign

**Keywords**: childhood cancer; aetiology; radon; magnetic fields; leukaemia classification

### INTRODUCTION

### Origin

Cancer in children under 15 years of age is rare, accounting for less than 1% of malignancies diagnosed each year in developed countries (Draper, 1995; Coleman et al, 1999). The types of cancer that occur in children are generally more responsive to chemotherapy than those in adults and, in recent years, improved treatment protocols have resulted in significant increases in survival for a wide range of diagnostic groups. These improvements have been particularly marked for leukaemia, which accounts for around a third of all childhood malignancies, and more than 70% of children diagnosed in the UK with this once rapidly fatal disease now survive into adulthood (Draper, 1995; Coleman et al, 1999). Cancer in children, nevertheless, remains a significant health problem. Of the 10 million children in the UK, around 1200 (one in every 8000) are diagnosed with the disease each year: about one child in every 600 developing it before their 15th birthday (Parkin et al, 1998; Coleman et al, 1999). Despite the marked improvements in treatment, about one in ten of all childhood deaths occurring after the first month of life is attributed to cancer and among 5– to 14-year-olds cancer is the second most common cause of death, after accidents (Botting and Crawley, 1995; Fear et al, 1999). Unfortunately, the marked advance in childhood cancer treatment seen over the last 20 years has not been matched by similar advances in knowledge of its aetiology and the causes of the majority of childhood cancers remain unknown (Draper et al, 1995; Miller et al, 1995; Little, 1999).

When, in 1990, this situation was discussed informally by members of the Radiation and Cancer Subcommittee of the United Kingdom Coordinating Committee on Cancer Research (UKCCCR) the Advisory Group on Non-Ionising Radiation of the National Radiological Protection Board (NRPB) and the Medical and Scientific Advisory Panel of the Leukaemia Research Fund (LRF), the only known causes apart from the hereditary factors that caused a few cases, were exposure to X-rays in utero or after birth, some types of chemotherapy given for a previous cancer, and infection with the Epstein–Barr virus. Many ideas had been floated, but often without distinguishing between the different types of the disease. Leukaemia had been the most extensively studied, although not always by sub-type. Among the many possible causes that had been suggested for leukaemia (or more specifically, in some studies for acute lymphoblastic leukaemia (ALL)) were: (1) exposure of the father to ionizing radiation before the child's conception (Gardner et al, 1990), or to a variety of other agents in the occupational and home environments

*Correspondence to:*
**Introduction**: R Doll, Clinical Trial Service Unit, Harkness Building, Radcliffe Infirmary, Oxford OX2 6HE, UK;
**Material and general methodology**: E Roman, LRF Centre for Clinical Epidemiology at Leeds University, Institute of Epidemiology, Margaret Smith Building, 30 Hyde Terrace, Leeds LS2 9LN, UK;
**Power frequency magnetic fields**: NE Day, Strangeways Research Laboratory, University of Cambridge, 2 Worts Causeway, Cambridge CB1 8RN, UK;
**Radon and terrestrial gamma radiation**: G Law, LRF Centre for Clinical Epidemiology at Leeds University, Institute of Epidemiology, Margaret Smith Building, 30 Hyde Terrace, Leeds LS2 9LN, UK;
**Diagnostic review and classification of solid tumours**: JM Birch, CRC Paediatric & Familial Cancer Research Group, Royal Manchester Children's Hospital, Stancliffe, Hospital Road, Manchester M7 4HA, UK;
**Leukaemia: biological samples and classification**: MF Greaves, LRF Centre, Institute of Cancer Research, Chester Beatty Laboratories, 237 Fulham Road, London SW3 6JB, UK;
**Molecular analysis of genetic susceptibility**: GM Taylor, Immunogenetics Laboratory, St Mary's Hospital, Hathersage Road, Manchester M13 6JH, UK

*Individuals who collaborated in producing this report and in obtaining the evidence on which it is based are listed at the end of the paper under the departments in which they worked.

(van Steensel-Moll et al, 1985; Arundel and Kinnier-Wilson, 1986; Lowengart et al, 1987); (2) exposure of the fetus transplacentally to a variety of N-nitroso compounds (Magee et al, 1976) or infection (Knox et al, 1983); and (3) exposure of the child postnatally to radon in house air (Henshaw et al, 1990) and to chloramphenicol given therapeutically (Shu et al, 1987). A history of viral infections (McKinney et al, 1987) and of infection arising from the mixing of urban and rural populations (Kinlen, 1988; Kinlen et al, 1990), or following a paucity of common infections in infancy (Greaves, 1988) and a deficit of immunizations (Kneale et al, 1986; McKinney et al, 1987) had also been associated with the disease.

Specific exposures suggested for specific sites or types of cancer other than ALL had, by 1990, included: chloramphenicol (Shu et al, 1987) and maternal drug use prior to or during pregnancy in relation to acute non-lymphoblastic leukaemia (Robison et al, 1989) barbiturates, and parental exposure to substances such as solvents (Gold and Gordis, 1978; Gold et al, 1979) and N-nitroso compounds (Preston-Martin et al, 1982) for central nervous system (CNS) tumours; and maternal exposures during or just prior to pregnancy to drugs (including recreational drugs and anti-convulsants), alcohol and hair colourants for neuroblastoma (Lipson and Bale, 1985; Kramer et al, 1987) and for Wilms' tumour (Bunin et al, 1987). It had also been suggested that Hodgkin's disease in children might arise as a rare sequel to infection with a common virus (Gutensohn and Cole, 1981; Gutensohn and Shapiro, 1982) and that children with rhabdomyosarcoma were less likely to be immunized than controls (Grufferman et al, 1982; Hartley et al, 1988).

For childhood cancer more generally, exposure to extremely low frequency electromagnetic fields from the passage of electricity (Wertheimer and Leeper, 1979) and the administration of vitamin K within the first week of life (Golding et al, 1990) had also been suggested as having a role.

### Objectives

It seemed, therefore, that it would be scientifically, socially and economically sensible to mount a national study of all types of childhood cancer on a large enough scale to provide sufficient data to test adequately the major hypotheses about the possible causes of such cancers, excluding only wide-ranging family studies of genetic factors.

After detailed discussions, five broad hypotheses were defined that the study should attempt to test. These were that childhood cancer might be caused by:

1. a child's exposure to ionizing radiation (natural or man-made) either during the mother's pregnancy or after birth
2. similar exposure to potentially hazardous chemicals, some of which have been associated with specific cancers in adult life
3. exposure of the parental germ cells to either radiation or hazardous chemicals before the child's conception
4. extremely low frequency electromagnetic fields post-natally, particularly in the case of brain cancers and leukaemia
5. in the special case of leukaemias and lymphomas, abnormal responses to one or more common infectious agents.

Two specific hypotheses were considered within the umbrella explanation of the fifth hypothesis. First, some subtypes (in particular common ALL) follow (i) paucity of infectious exposure in infancy, and (ii) late or delayed exposure shortly before the onset of symptoms (3–12 months prior to diagnosis). Secondly, the risk of leukaemia (and possibly lymphoma) is increased in children in rural areas of marked population mixing. In either case, the leukaemia may also be associated with genetic factors that influence the immune response – in particular, HLA class II alleles or haplotype.

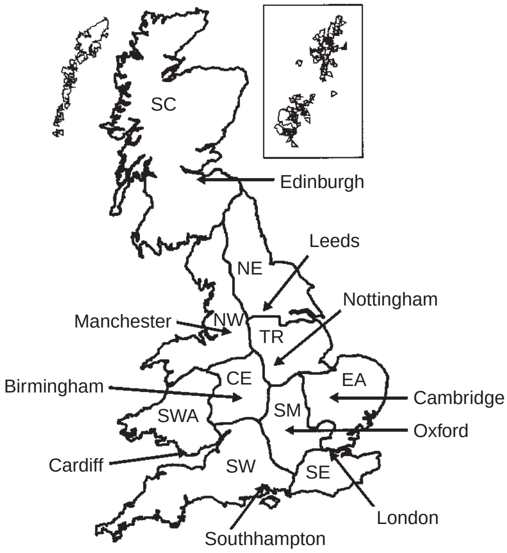

**Figure 1** Regional study centres. SC, Scotland; NE, North-east; NW, North-west; TR, Trent; CE, Central; EA, East Anglia; SM, South Midlands; SWA, South-Wales; SW, South-west; SE, South-east

### Organization

A management committee was consequently set up comprising epidemiologists, paediatric oncologists, laboratory scientists and a representative of the NRPB, the members of which planned the conduct of the study under the aegis of the UKCCCR. The committee divided England & Wales into the nine regions shown in Figure 1, each of which became the responsibility of a leading epidemiologist who, in conjunction with local paediatric oncologists and the help of the UK Children's Cancer Study Group, arranged for the collection of the necessary information.

A similar study was initiated independently at about the same time in Scotland under the aegis of the Scottish Department of Health. Early contacts enabled it to be agreed that similar data would be collected in much the same way both in Scotland and in England & Wales, that the principal Scottish investigator would join the Management Committee and that the principal results obtained in all three countries would be pooled for central analysis. Consequently Scotland became, in effect, the tenth region in a study, named the United Kingdom Childhood Cancer Study (UKCCS), that covered the whole of the UK, apart from Northern Ireland.

With the rare exceptions described in the section on General Methodology, the study was planned to include all children up to 15 years of age registered with a Family Health Services Authority (FHSA) in England & Wales and with a Health Board (HB) in Scotland who were diagnosed as having cancer. Case accrual began in Scotland on 1 January 1991 and in England & Wales on 1 April 1992 and was planned to continue until at least 1000 cases

 © 2000 Cancer Research Campaign

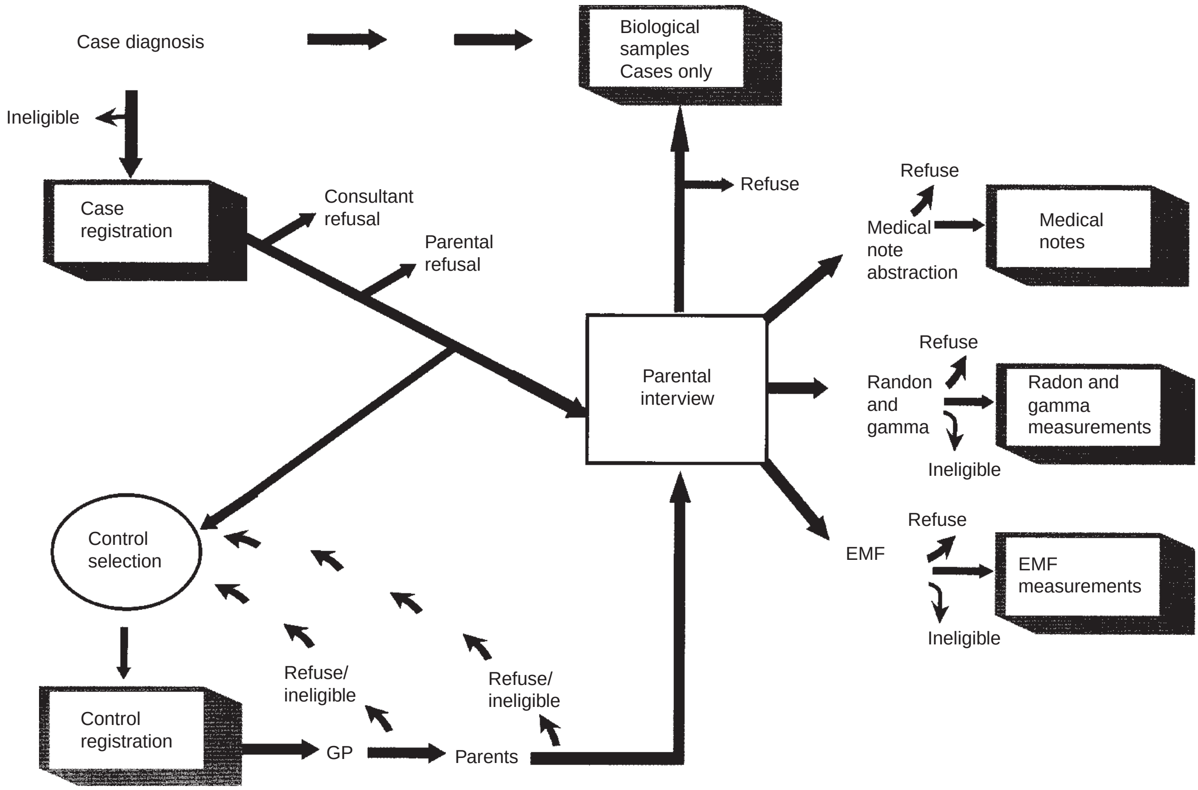

**Figure 2** Study organization and main components. EMF, extremely low frequency electromagnetic fields of ALL had been studied, which it was thought should take about 4 years. In the event, accrual ceased in different areas at different dates between December 1994 and December 1996. For each child with cancer, two controls were sought matched on sex and month and year of birth from the same FHSA or HB list as that on which the child with cancer had been registered before the cancer was diagnosed. Altogether the study included 1461 children with acute lymphoblastic leukaemia, 2377 children with other types of cancer and 7629 control children (47 children with cancer having only one control).

Details of the information obtained and how it was collected are described in sections on materials and general methodology, electromagnetic fields, radon and terrestrial gamma radiation, classification of solid tumours, biological samples and classification of leukaemia, and genetic susceptibility.

### MATERIALS AND GENERAL METHODOLOGY

The ten UKCCS data collection centres (Figure 1) were responsible for all aspects of regional study organization including the day-to-day running of the study, obtaining permission from local ethical committees and liaising with appropriate health care professionals. An outline of a typical organizational chart for data collection is given in Figure 2.

A common protocol was agreed by the Management Committee at the outset of the study. The need to respond at a local level to prevailing circumstances – such as specific requests by local ethical committees etc. – resulted in a certain amount of regional variation. The main departures from the protocol, together with basic regional information, are listed by region in Table 1.

### Subjects

In England & Wales, the UKCCS study population was defined as children (0–14 years) registered with one of the 98 FHSAs. Under the National Health Service, all General Practitioners (GPs) and their patients are registered with their local FHSA. This comprises around 98% of the total population (RCGP, 1987). In Scotland, where the system is similar but independent, the study population was defined as children registered with one of the 15 Health Boards (HBs) and has been described separately (McKinney et al, 1995).

### Cases

The study began in Scotland on 1 January 1991 and on 1 April or 1 September 1992 in the regions of England & Wales (see Table 1). Following these start dates, children registered with an FHSA/HB who were diagnosed either with a confirmed malignancy or with any tumour of the CNS, as defined in the classification scheme devised by Birch and Marsden (1987), were potentially eligible for the study. In Scotland, case accrual ceased in December 1994, and in England & Wales it was restricted in all regions to patients with leukaemia and non-Hodgkin's lymphoma throughout 1995, and leukaemia alone throughout 1996.

Each regional centre was responsible for ensuring the completeness of ascertainment of cases diagnosed in residents within their boundaries. The majority of cases were notified directly from regional treatment centres by paediatric oncologists belonging to the UKCCSG (Mott et al, 1997). In other hospitals, and certain specialist units that treated adults as well as children, individually

**Table 1** Case accrual and control selection by study region

| Study region | Accrual dates (no. of children studied) | | | Control selection |
|---|---|---|---|---|
| | Leukaemia | Non-Hodgkin's lymphoma | Other cancers | |
| Central | 09/92–12/96 (153) | 09/92–12/95 (23) | 09/92–12/94 (140) | As protocol |
| East Anglia | 04/92–12/96 (172) | 04/92–12/95 (20) | 04/92–12/94 (160) | Note a |
| North East | 04/92–12/96 (194) | 04/92–12/95 (29) | 04/92–12/94 (228) | As protocol |
| North West | 09/92–12/96 (220) | 09/92–12/95 (30) | 09/92–12/94 (249) | Note d |
| Scotland | 01/91–12/94 (156) | 01/91–12/94 (22) | 01/91–12/94 (272) | Note b |
| South East | 04/92–03/96 (225) | 04/92–12/95 (32) | 04/92–12/94 (281) | As protocol |
| South Midlands | 04/92–12/96 (170) | 04/92–12/95 (21) | 04/92–12/94 (150) | As protocol |
| South Wales | 09/92–12/96 (53) | 09/92–12/95 (9) | 09/92–12/94 (38) | Note c |
| South West | 04/92–12/96 (234) | 04/92–12/95 (24) | 04/92–12/94 (187) | Note d |
| Trent | 04/92–12/96 (159) | 04/92–12/95 (21) | 04/92–12/94 (166) | Note e |

Notes: [a]For cases diagnosed before 9/93 FHSA records ordered by date of birth and same sex and controls with closest birth-date selected. [b]As in protocol but controls selected by the health boards. [c]Each pair of controls matched on sex and month of birth (± 3 months) randomly selected from each of two randomly selected GP lists. [d]One FHSA withheld permission for data access. Controls selected as in note c. [e]For cases diagnosed before 4/96, controls selected as in note a.

tailored referral systems were put in place. In addition, cross-checks were made against regional cancer registries and against the National Registry of Childhood Tumours (NRCT) (Stiller et al, 1995).

Detailed diagnostic information was obtained from multiple sources. For leukaemias, the principal diagnostic sources were the Medical Research Council's treatment trials (MRC, 1997, 1998). Information about leukaemic children who were not enrolled in trials was obtained from one of three sources: the UKCCSG, the NRCT, or the individual consultant treating the child. In addition, cytogenetic data on trial and non-trial patients were obtained from the Leukaemia Research Fund's cytogenetics database held at the Royal Free Hospital (Moorman et al, 1996), and molecular diagnostic information on patients with ALL was provided by the central reference laboratory for UKCCS pretreatment samples at the Leukaemia Research Fund Centre, Institute of Cancer Research. To obtain reliable information about the diagnosis of cancers other than leukaemia, a histopathology review database was specially created for the purposes of the study. Detailed information about this database, and the diagnostic verification procedures used, are given later.

### Controls

Each case child was individually matched with two control children of the same sex, date of birth and region of residence. The mechanism by which this was achieved varied slightly from one region to another (Table 1).

Throughout the majority of English study regions controls, matched on sex and age (month and year of birth), were randomly selected from the same FHSA list as their corresponding case. This was achieved by obtaining computerized lists of children registered with each FHSA on 1 January and 1 July each year: the potential controls being randomly selected from the list on which the case child had appeared immediately prior to the diagnosis of the cancer. When the case child was less than 1 year old, however, controls were chosen from the first FHSA list on which the case appeared. Because of computing difficulties, two English study regions did not randomly select potential controls from within the same birth month as the corresponding case. Instead, the FHSA downloads were sorted in date of birth order and controls with the closest birthdates to the case selected (hence, most controls were born on the same day as their corresponding case) (Table 1).

In Scotland, the system was similar to the main method described above except that the Health Boards randomly selected the controls (McKinney et al, 1995). Unfortunately seven of the eight Welsh FHSAs and one English FHSA declined to participate in the study. For each case diagnosed within their boundaries the corresponding control selection had two stages: two GPs were randomly selected and approached and, if they agreed, potential controls (of the same sex and born within 3 months of the corresponding case) were randomly selected from their practice lists.

Whatever procedure was used to identify potential controls, the GPs of the first two identified were approached and, with their permission, the parents of the children were contacted and asked to participate in the study. When the GP refused permission to contact, or the parents refused to participate, another control was selected by the same method, and so on until two control families participated (Figure 2). All details of control replacements were logged, and details of non-participating controls retained (see Registration below).

 *© 2000 Cancer Research Campaign*

Ethnicity was ignored during control selection, but a third control family was, where possible, interviewed in place of the second family when the parents of both control children were of a different ethnic group to the parents of the case child. In most regions this additional selection was possible only when the case was white and the two interviewed controls were not.

### Ineligible children

Children (cases and controls) were ineligible if they had been born outside Great Britain or had a prior malignancy. For the purposes of the study, all control children were assigned a 'pseudo-diagnosis' date that coincided with the exact age at which their corresponding case was diagnosed. Children who themselves, or whose parents, were resident outside Great Britain in the 3 months leading up to diagnosis/pseudo-diagnosis were considered ineligible. For ethical reasons, children in residential local authority care at diagnosis/pseudo-diagnosis (< 1% of the total childhood population) were also excluded from the study.

### Data collection

### *Registration*

At registration the date of birth, sex, initial diagnosis and home address of all eligible cases diagnosed in the regions were recorded, regardless of whether their families subsequently participated in the study. Controls were selected only for participating cases. All control children whose GP was approached were registered in the system, regardless of whether their GP/parents agreed to participate. The system was designed so that the origins of all controls attached to a case were known, the key fields being:

1. Eligibility: yes or no
2. Identification number: chronological order in the selection sequence
3. Replacement number: identification number of the control replaced
4. Choice number: 1st, 2nd, etc. (this variable is less than the identification number when new controls replace ineligible controls).

For those children whose families were recruited, the address at diagnosis/pseudo-diagnosis was recorded at registration. For those whose families did not participate, the initial contact address supplied by the treating hospital (cases) or listed on the FHSA/HB database (controls) was recorded.

### *Participation*

Eighty-seven per cent of the parents of the 4433 case children agreed to be interviewed but only 64% of the 11 987 potentially eligible control children. The numbers not participating for different reasons are shown in Figure 3.

A few (3% of the cases and less than 1% of the controls) were not approached. This occurred when the mother (biological or non-biological) of the child had died and no suitable surrogate could be found, when the parents did not speak English and for whom no suitable translator could be found, or when the family emigrated shortly after diagnosis/pseudo-diagnosis. For both cases and controls, parental refusal to participate was the main reason an interview was not secured, accounting for 279 (47%) of the 595 non-interviewed cases and 2625 (60%) of the 4358 non-interviewed controls. Refusals by medical practitioners to allow their patients to participate in the study was also important: permission to approach families was not given by the consultants treating 116 cases and by the GPs of 1012 controls. For controls, another important factor for not securing an interview was 'failure to trace': the address held by the GP of 648 (5% of the total and 15% of the not interviewed) was incorrect, and the family's correct address was not traceable.

### *Parental interview*

Mothers and fathers were interviewed using separate, but similar, questionnaires. The parent(s) or guardian(s) with whom the child was living at the time of diagnosis/pseudo-diagnosis were approached and interviewed first, regardless of whether they were the biological or non-biological parent(s). In the case of the latter, permission was then sought to contact and interview the biological parent(s).

A trained interviewer using a structured questionnaire interviewed all participating parents. Telephone interviews were conducted only when face-to-face interviews could not be arranged. When one parent (usually the father) could not be contacted, the available parent (usually the mother) was asked to act as a surrogate.

Full residential and occupational histories, including specific information about occupational exposures and individual housing characteristics, were recorded for each parent. To improve the quality of these data, a form asking parents to list the places where they had lived and the jobs they had had was sent out in advance of the interview. At interview, mothers and fathers were also asked about their own health, social habits and illnesses in their families. Additional sections on pregnancies and on the index child's health, schooling and social history were incorporated into the mother's questionnaire.

On completion of the interview, parents were asked whether they were willing to be contacted again. In particular, consent was sought for their participation in both the ionizing radiation (radon and gamma) and the non-ionizing radiation (EMF) components of the study (Figure 2). Signed agreement was also requested for blood samples of the cases to be taken at a later date and for medical and other records to be accessed.

### *Medical note abstractions*

With the interviewee's and their GP's permission, the GP notes of index children and their biological parents were abstracted onto

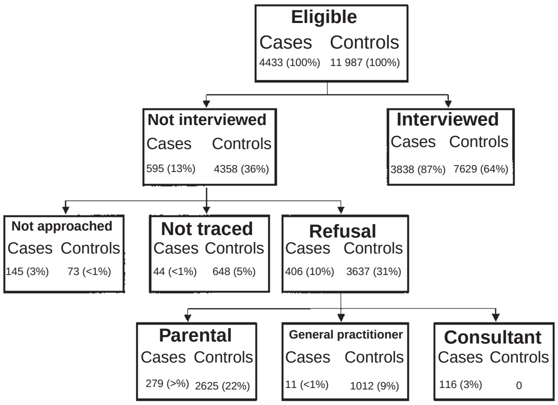

**Figure 3** Numbers of eligible and participating families and numbers not participating for different reasons

**Table 2** Characteristics of interviewed cases

| | Leukaemia | | Lymphoma | | | | |
|---|---|---|---|---|---|---|---|
| | **Acute lymphoblastic** *n* (%) | **Other** *n* (%) | **Non-Hodgkin's** *n* (%) | **Hodgkin's** *n* (%) | **Central nervous system tumours** *n* (%) | **Other cancers**[a] *n* (%) | **All cancers** *n* (%) |
| Total | 1461 (100) | 275 (100) | 231 (100) | 118 (100) | 686 (100) | 1067 (100) | 3838 (100) |
| Diagnostic information source | | | | | | | |
| Treatment trial | 1313 (89.9) | 188 (68.4) | – | – | – | – | 1501 (39.1) |
| Histopathology register[b] | – | – | 231 (100) | 118 (100) | 686 (100) | 1067 (100) | 2102 (54.8) |
| Other | 148 (10.1) | 87 (31.6) | – | – | – | – | 235 (6.1) |
| Sex | | | | | | | |
| Boys | 820 (56.1) | 151 (54.9) | 167 (72.3) | 85 (72.0) | 345 (50.3) | 586 (54.9) | 2154 (56.1) |
| Girls | 641 (43.9) | 124 (45.1) | 64 (27.7) | 33 (28.0) | 341 (49.7) | 481 (45.1) | 1684 (43.9) |
| Age at diagnosis (years) | | | | | | | |
| 0 | 50 (3.4) | 38 (13.8) | 0 (0.0) | 0 (0.0) | 51 (7.4) | 177 (16.6) | 316 (8.2) |
| 1–4 | 795 (54.4) | 94 (34.2) | 53 (22.9) | 9 (7.6) | 215 (31.3) | 438 (41.0) | 1604 (41.8) |
| 5–9 | 394 (27.0) | 58 (21.2) | 71 (30.7) | 36 (30.5) | 258 (37.6) | 229 (21.5) | 1046 (27.3) |
| 10–14 | 222 (15.2) | 85 (30.9) | 107 (46.3) | 73 (61.9) | 162 (23.6) | 223 (20.9) | 872 (22.7) |
| Median | 4.4 | 5.4 | 9.5 | 11.9 | 6.4 | 3.8 | 5.0 |
| Ethnicity | | | | | | | |
| White[c] | 1224 (83.8) | 216 (78.5) | 197 (85.3) | 96 (81.4) | 586 (85.4) | 896 (84.0) | 3215 (83.8) |
| Year of birth | | | | | | | |
| ≤ 1984–1985 | 313 (21.4) | 101 (36.7) | 135 (58.4) | 89 (75.4) | 277 (40.4) | 331 (31.1) | 1246 (32.5) |
| 1986–1990 | 647 (44.3) | 63 (22.9) | 79 (34.1) | 28 (23.7) | 274 (39.9) | 350 (32.8) | 1441 (37.5) |
| ≥ 1991 | 501 (34.3) | 111 (40.3) | 17 (7.3) | 1 (0.8) | 135 (19.6) | 386 (36.1) | 1151 (30.0) |
| Median | 1989 | 1988 | 1984 | 1981 | 1987 | 1989 | 1988 |

[a]Peripheral neural tumours, retinoblastomas, soft tissue sarcomas, carcinomas, renal tumours, hepatic tumours, bone tumours, Langerhan's cell histiocytosis germ cell tumours, and other lympho-reticular and unspecified tumours. [b]Specially created for UKCCS (reference histopathology section). [c]Both biological parents White.

specially designed forms: information being collected about past illnesses, prescribed drug usage, vaccinations received and other medical procedures. Similarly, details about the mother's pregnancy with the index child were transferred from her obstetric notes and the index baby's neonatal notes (where they existed) to structured obstetric abstraction forms. More information about the variables included on these forms is given elsewhere (Roman et al, 1997; McKinney et al, 1999).

### *Ionizing and non-ionizing radiation*

The arrangements made for measuring the children's exposure to electromagnetic fields and to radon and terrestrial gamma radiation are described on pp. 1087–1090.

### *Biological samples*

After obtaining parental permission, pretreatment blood samples were sought from all children with cancer, and marrow samples from all with leukaemia and lymphoma. Following interview, blood samples were also sought from the case children's first-degree relatives or, when appropriate, from the children during remission. The number of samples collected and what was done with them are described later.

### Data management and census linkage

Registration and interview data were coded and entered into computers in the regional centres using the database management system FoxPro version 2.6 (FoxPro, 1991). Parental occupational histories were coded according to the Office of Population Censuses and Surveys 1990 schemes (Office of Population Censuses and Statistics, 1990; Central Statistics Office, 1992; Office of National Statistics Office, 1998), and illnesses according to the International Classification of Diseases tenth revision (WHO, 1990, 1992). On completion of regional checking, the data were further scrutinized, merged and transferred to Microsoft SQL (structured query language) Server version 6.5 at the Study's national centre in Leeds. At the same time, the regionally collected case registration data were linked to various national diagnostic and biological databases, and consequent discrepancies resolved, as described later.

With a view to linking to areal information derived from the 1991 census (Census, 1991), particular attention was paid to collecting valid postcoded addresses for certain key points in time. For participants, these included the homes the child lived in at the time of diagnosis/pseudo-diagnosis and at the time of birth. For non-participants the only available address was the 'contact' address, as held by the FHSA/HB or by the GP. The postcodes of all addresses were checked and, where necessary, automatically reassigned using QuickAddress (1999). About 5% of all addresses could not be resolved in this way, and postcodes were manually allocated. Each address was then linked to one of the 108 336 enumeration districts (EDs) in England & Wales or 38 084 output areas (OAs) in Scotland using dictionaries of correspondence between postcode and census units. The establishment of this linkage enabled aggregate information derived from the census to be used as an areal indication of factors such as wealth and population density.

The 'deprivation' index used in the present report is similar to that developed for a national study of the geographical distribution of childhood leukaemia (Draper et al, 1991). For each ED in England & Wales and OA in Scotland, the following proportions were calculated:

1. households without a car
2. overcrowded households (more than one person per room)

 *© 2000 Cancer Research Campaign*

**Table 3** Interviewed controls per interviewed case by control choice

| Number of first choice controls per case | Number (%) of interviewed controls per case | | |
|---|---|---|---|
| | One | Two | Total |
| None | 23 (0.6) | 458 (11.9) | 481 (12.6) |
| One | 24 (0.6) | 1573 (41.0) | 1597 (41.6) |
| Two | – | 1760 (45.9) | 1760 (45.9) |
| Total | 47 (1.2) | 3791 (98.8) | 3838 (100.0) |

3. persons unemployed: the denominator being persons who were 'economically active' (over 16 years of age, with a potential to be gainfully employed or in receipt of unemployment benefit).

In simple terms, following a series of standard transformation procedures, the standard deviation from the mean of each of the three variables were summed for each area (Townsend et al, 1988). The 146 420 small areas were then ranked by the resulting 'deprivation' index (low values indicating relative affluence and high values deprivation) and divided into seven equal categories (categories one to six each containing 20 917 areas and category seven 20 918). To enable the contribution of the three components of the index to be examined separately, each component proportion was divided into seven categories (as for the deprivation index). For overcrowding, areas with zero proportions formed the bottom category, with the remaining areas being divided into six equally sized groups.

### Characteristics of participants

The characteristics of the 3838 children diagnosed with cancer whose families participated in the study are summarized in Table 2.

Detailed diagnostic information about 1313 (90%) children with ALL and 188 (68%) children with other forms of leukaemia was obtained from MRC treatment trials (Table 2). Confirmatory diagnostic data on the 235 children with leukaemia who were not entered into a trial and the 2102 children with other forms of cancer were obtained from a variety of sources (see pp. 000 and 000)

There are more boys than girls in each malignancy group. The largest sex difference was for lymphomas, where 72% of those diagnosed were male, and the smallest sex difference was for tumours of the CNS where just over 50% were male (Table 2). The age-distributions also varied by cancer type. Nearly 60% of the 1461 children with ALL were diagnosed before their 5th birthday, the median age at diagnosis being 4.4 years. By contrast, around 60% of the children with Hodgkin's disease were diagnosed after their 10th birthday, the median age at diagnosis being 11.9 years.

**Table 4** Characteristics of the interview

| | Mother[a] | | Father[a] | |
|---|---|---|---|---|
| | Case *n* (%) | Control *n* (%) | Case *n* (%) | Control *n* (%) |
| Total interviews | 3838 (100) | 7629 (100) | 3679 (100) | 7135 (100) |
| Biological parent | | | | |
| Yes | 3818 (99.5) | 7600 (99.6) | 3596 (97.7) | 7010 (98.2) |
| No | 20 (0.5) | 29 (0.4) | 83 (2.3) | 125 (1.9) |
| Mode of interview | | | | |
| Personal | 3772 (98.2) | 7550 (99.0) | 3153 (85.7) | 5143 (72.1) |
| Surrogate | 66 (1.7) | 79 (1.0) | 526 (14.3) | 1992 (27.9) |
| Marital status | | | | |
| Married/co-habiting | 3302 (86.0) | 6519 (85.5) | 3243 (88.1) | 6334 (88.8) |
| Separated/divorced | 303 (7.9) | 650 (8.5) | 239 (6.5) | 454 (6.4) |
| Other/not known | 233 (6.1) | 460 (6.0) | 197 (5.4) | 347 (4.9) |
| Housing tenure | | | | |
| Owner | 2566 (66.1) | 5433 (71.2) | 2473 (67.2) | 5142 (72.1) |
| Tenant | 1156 (30.1) | 1992 (26.1) | 907 (24.7) | 1407 (19.7) |
| Other/not known | 116 (3.0) | 204 (2.7) | 299 (8.3) | 586 (8.2) |
| Age (years) | | | | |
| < 29 | 1153 (30.0) | 2082 (27.3) | 691 (18.8) | 1204 (16.9) |
| 30–39 | 2039 (53.1) | 4189 (54.9) | 1912 (52.0) | 3713 (52.0) |
| > 40 | 644 (16.8) | 1352 (17.7) | 1045 (28.4) | 2165 (30.3) |
| Not known | 2 (<0.1) | 6 (0.1) | 31 (0.9) | 53 (0.7) |
| mean + standard deviation | 33.6+6.4 | 34.0+6.3 | 36.4+7.2 | 36.7+7.1 |
| Social class[b] | | | | |
| I | 43 (1.1) | 99 (1.3) | 253 (6.9) | 580 (8.1) |
| II | 497 (12.9) | 1291 (16.9) | 898 (24.4) | 1818 (25.5) |
| III NM | 558 (14.5) | 1373 (18.0) | 272 (7.4) | 585 (8.2) |
| III M | 83 (2.2) | 216 (2.8) | 887 (24.1) | 1777 (24.9) |
| IV | 338 (8.8) | 815 (10.7) | 406 (11.0) | 787 (11.0) |
| V | 107 (2.8) | 230 (3.0) | 107 (2.9) | 167 (2.3) |
| Armed forces | – | – | 40 (1.1) | 62 (0.9) |
| Not gainfully employed | 2212 (57.6) | 3605 (47.3) | 816 (22.2) | 1359 (19.0) |

[a]Not more than two parents per child are included, biological parents taking precedence if both biological and other parents were interviewed. [b]Based on occupation at the time of diagnosis.

**Table 5** Areal deprivation characteristics of interviewed cases and controls

| | Deprivation index[a] | | Components of the deprivation index: Not gainfully employed[a] | | Overcrowding[b] | | Car ownership[b] | |
|---|---|---|---|---|---|---|---|---|
| | Cases *n* (%) | Controls *n* (%) | Cases *n* (%) | Controls *n* (%) | Cases *n* (%) | Controls *n* (%) | Cases *n* (%) | Controls *n* (%) |
| Total | 3838 (100) | 7629 (100) | 3838 (100) | 7629 (100) | 3838 (100) | 7629 (100) | 3838 (100) | 7629 (100) |
| Least deprived | 531 (13.8) | 1153 (15.1) | 502 (13.1) | 1041 (13.7) | 791 (20.6) | 1584 (20.8) | 424 (11.1) | 716 (9.4) |
| | 602 (15.7) | 1212 (15.9) | 592 (15.4) | 1295 (17.0) | 621 (16.2) | 1227 (16.1) | 474 (12.4) | 925 (12.1) |
| | 589 (15.4) | 1160 (15.2) | 580 (15.1) | 1245 (16.3) | 551 (14.4) | 1129 (14.8) | 551 (14.4) | 1057 (13.9) |
| | 557 (14.5) | 1155 (15.1) | 564 (14.7) | 1096 (14.4) | 491 (12.8) | 994 (13.0) | 570 (14.9) | 1158 (15.2) |
| | 504 (13.1) | 1037 (13.6) | 532 (13.9) | 1042 (13.7) | 490 (12.9) | 980 (12.9) | 591 (15.4) | 1269 (16.6) |
| | 510 (13.3) | 993 (13.0) | 518 (13.5) | 958 (12.6) | 455 (11.9) | 956 (12.5) | 612 (16.0) | 1221 (16.0) |
| Most deprived | 545 (14.2) | 919 (12.1) | 550 (14.3) | 952 (12.5) | 439 (11.4) | 759 (10.0) | 616 (16.1) | 1283 (16.8) |
| $\chi^2$ (*P*-value) | 13.4 (0.04) | | 15.4 (0.02) | | 6.9 (0.33) | | 8.8 (0.19) | |

[a]All UK enumeration districts divided into seven equally sized categories. [b]The lowest group contains all zero counts, with the rest divided into six equally sized categories.

**Table 6** Percentage of first choice interviewed and non-interviewed controls distributed by areal deprivation[a]

| | | Total (*n* = 7632) | Interviewed: Yes (*n* = 5530) | No (*n* = 2102)[b] |
|---|---|---|---|---|
| Least deprived | 1 | 14.0 | 15.9 | 8.9 |
| | 2 | 14.3 | 16.1 | 9.8 |
| | 3 | 13.7 | 14.9 | 10.6 |
| | 4 | 14.5 | 15.2 | 12.6 |
| | 5 | 13.9 | 13.7 | 14.7 |
| | 6 | 14.3 | 12.8 | 18.3 |
| Most deprived | 7 | 15.2 | 11.4 | 25.2 |

[a]see Methods section for definition; [b]37 non-interviewed first-choice controls had missing addresses.

Although 3791 (99%) cases had two interviewed controls, 47 (1%) had only one because a second interview was not secured before the end of the study (Table 3). Further controls were selected if those first selected did not participate. Both participating controls of 1760 (46%) cases were first choices, as was one of the two participating controls of 1597 (42%) cases; the remaining 481 (13%) cases had no first choice controls interviewed.

For each child enrolled in the study, up to four parents (biological and non-biological) were interviewed, with 121 cases (3%) and 182 controls (2%) having three or more participating parents. Overall, the biological mothers of 3818 (99%) cases and 7600 (99%) controls were interviewed. The corresponding proportions for biological fathers were slightly lower, at 3596 (94%) for cases and 7010 (92%) for controls.

The characteristics of the parents interviewed are summarized in Table 4. If both biological and non-biological parents were interviewed, the data for biological parents are given precedence. It should be noted that mothers and fathers were not necessarily living together at the time of the interview.

There are a number of differences between cases and controls with respect to the characteristics listed in Table 4. For fathers, but not for mothers, surrogate interviews were twice as common among controls as among cases: information on about 28% of control fathers compared to 14% of case fathers being supplied by someone else ($P < 0.05$). For both cases and controls, the surrogate in over 99% of instances was the child's mother. The time interval between diagnosis/pseudo-diagnosis and interview varied markedly between cases and controls: the medians being 5.8 months and 14.0 months for case and control mothers respectively.

At the time of interview, parents of control children were more likely than parents of case children to be married (but not necessarily to each other) and to own their own home. At the time of the child's diagnosis/pseudo-diagnosis, participating control parents were, on average, significantly older and of a higher social class (as assigned on the basis of their own occupation). With respect to the latter, the largest difference occurred in the proportion who were not employed at the time of interview: for cases and controls the proportions being 58% versus 47% and 22% versus 19% for mothers and fathers respectively (Table 4).

More information about the socio-economic differences between participating cases and controls is given in Table 5, which shows the census-derived findings for areal deprivation assigned on the basis of the child's address at the time of diagnosis/pseudo-diagnosis. Overall, the data indicated that participating control families tended to live in more affluent areas than case families: there being less unemployment and overcrowding and fewer homes without access to a car. Interestingly, in agreement with the findings for social class assigned on the basis of parental occupation (Table 4), the only significant heterogeneity was for the proportion unemployed within an area.

That these observations are principally due to participation bias is supported by the findings presented in Table 6, which compares the deprivation distributions of interviewed and non-interviewed first-choice controls. Within this group there are marked differences between the 5530 who were interviewed and the 2102 who were not, the latter being far more likely to live in deprived areas than the former. Importantly, the deprivation distribution of the 7632 first-choice controls was not significantly different from that of the 3838 cases distribution shown in Table 5.

### Discussion

The response from case families was good, with 3838 (87%) of the 4433 eligible cases identified participating. Direct assessment of

 *© 2000 Cancer Research Campaign*

the completeness of UKCCS case ascertainment for the years covered by the study is not straightforward. Ineligible case children (e.g. those who were born abroad) were not registered in the UKCCS, and data on all eligible diagnoses were collected in all regions only for the years 1993 and 1994. Broad comparison with national data for 1981–1990 suggests, however, that the proactive case ascertainment methods used in the study were efficient. Further, the age and sex distributions of the diagnoses included in the UKCCS are reassuringly similar to those reported for Britain in the 10-year period 1981–1990 (Parkin et al, 1998).

Participation was not as high for control families as for case families: parental interviews being secured for 7629 (64%) of control families compared with 3838 (87%) of case families. The most important reason for not having an interview was parental refusal, with 2625 (22%) control parents and 279 (7%) case parents refusing to participate in the study. In addition to refusals, 5% of the selected control families did not live at the address supplied by the medical authorities and could not be traced to any other address. This 5% error rate for addresses is in line with other reports (RCGP, 1987; Page, 1991; Roberts et al, 1995).

Like all case–control studies that have relied on the recruitment of individuals for collection of information, the UKCCS findings have to be carefully evaluated in the light of participation bias. Participation bias is introduced when those who respond to a study differ in important respects from those who do not, the consequences in case–control studies invariably being exacerbated by the fact that cases and controls are not equally affected. The design of the UKCCS allows the potential effects of participation bias to be examined in more detail than has been possible in other large-scale case–control studies of childhood cancer (Kleinerman et al, 1997; McBride et al, 1999): the census linkage enabling the characteristics of areas in which participators lived to be compared with those of non-participators. The findings from this areal analysis confirm that the socio-economic differences between interviewed cases and controls arose because controls categorized as deprived were less likely to participate than controls who were more affluent.

### POWER FREQUENCY MAGNETIC FIELDS: DESIGN CONSIDERATIONS

When the UKCCS was initiated, previous studies had suggested that a possible excess of childhood malignancy, particularly leukaemia and brain cancer, might be associated with above average levels of exposure to 50 or 60 Hz electromagnetic fields. The evidence, however, was inconclusive and further studies based on appropriate measurements were needed to elucidate the nature of the relationship between 50 and 60 Hz electromagnetic fields and childhood malignancy (Advisory Group on Non-ionising Radiation, 1992).

We describe here the design of the part of the UKCCS relevant to exposure from 50 Hz magnetic fields (and harmonics below 800 Hz) generated by the distribution and use of electricity. Residential 50 Hz electric fields were the subject of a pilot study embedded in the magnetic field study and will form the subject of a separate paper.

The primary hypothesis, based on the results of the studies reviewed by the NRPB's Advisory Group on Non-ionising Radiation (1992, 1994), was that risk for some childhood malignancies is positively associated with the average magnetic field level to which a child is exposed in the year before diagnosis. In particular, average exposures greater than 0.2 microtesla (μT) in the year before diagnosis would confer greater risk for (i) all leukaemias; (ii) ALL and (iii) CNS tumours, than average exposure less than 0.1 μT. A further hypothesis is that risk for each of the above diagnostic categories increases steadily across the range of dose from less than 0.1 μT to greater than 0.4 μT.

During the course of the UKCCS, but before any analyses were undertaken, further measurement-based studies had refined the hypothesis, suggesting that if any risk did exist it was typically above the 95th percentile of the distribution in the controls of these studies (Linet et al, 1997; Michaelis et al, 1997; Dockerty et al, 1998; McBride et al, 1999).

### Assessment of EMF exposure

For the purposes of this study EMF exposure is defined as the time-averaged estimate of whole body average magnetic flux density as derived from the true root-mean-square (RMS) measurements made in three orthogonal directions. It is a physical entity for which a 'gold standard' measure is theoretically available, at least for the average field over a year – defined as the exposure of interest in this study. Personal monitors worn for extended periods of time would provide an approximation to the annual average exposure of an individual. Their use in this study was ruled out on two grounds: firstly, exposure measured by personal monitor after a diagnosis of malignancy cannot be taken as a measure of predisease exposure, since the lifestyle will change; secondly, the cost for the numbers involved would be excessive.

The measurement protocols in the UKCCS were based on a pilot study conducted by NRPB. A total of 51 children from the North East of the Leeds metropolitan area, of a similar age and sex distribution to all children diagnosed with cancer in the UK, wore a magnetic field monitor for 48 h. Contemporaneous measures of 50 Hz magnetic flux density were taken in the family room (2 h), at the centre of up to five rooms in the home (5 min per room), on the centre of the bed (2 × 5 min, separated by 2 h) and at the bedside (48 h). Measurements were made also in the classroom (5 × 5 min) of those children attending a school or nursery.

On the basis of this pilot study, the 48-h personal exposure of children was moderately well correlated with combined short-term measurements made in the bedrooms and family rooms. The correlation was improved when the assessment of exposure included school measurements and/or residential measurements over 48 h ($r \geq 0.8$). It was concluded that household assessment could provide an adequate measure of an individual's exposure, provided that schools were also measured when relevant.

The pilot study indicated that a restricted set of measurements would classify, with acceptable sensitivity and specificity, an individual into the lowest 90% of exposure. For exposures in the top 10%, more extensive measurements would be required, to give a more precise exposure estimate. Resources, however, were not available for extensive measurements in the households of all cases and controls. A two-phase approach was therefore adopted for the UKCCS.

In the first phase, data were collected for a series of matched case–control pairs with a single control per case. Information on EMF exposures was gathered from five different sources (i) specified measures in the child's home; (ii) specified measures in schools or other institutions, such as purpose built nursery schools, attended by the child; (iii) a questionnaire on electrical appliances

© 2000 Cancer Research Campaign 

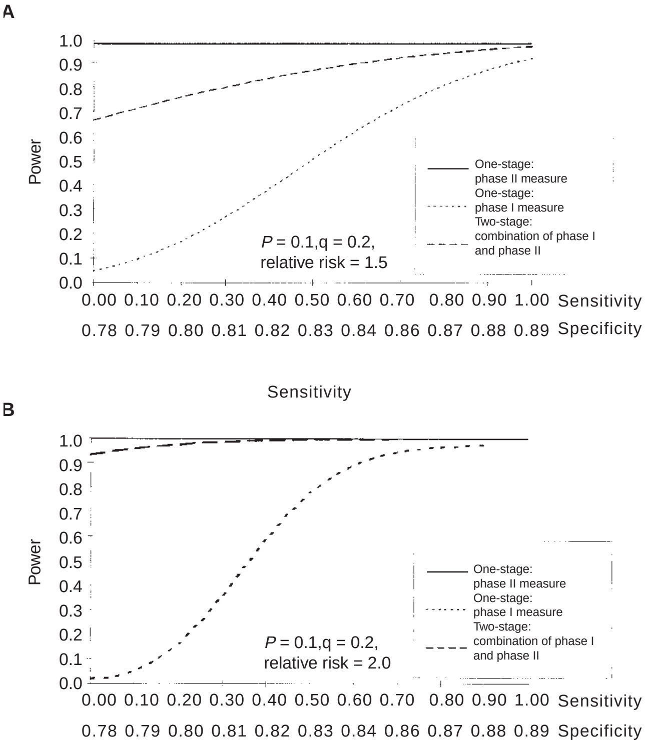

**Figure 4** Comparison of three sampling strategies: $n = 500$, $P$ = proportion of controls exposed (phase II measure), q = proportion classified as exposed by the surrogate (phase I measure)

in the home; (iv) the proximity and type of overhead power lines nearby from an external sources questionnaire (ESQ) and (v) electricity company databases of historical load data and other operating characteristics.

In the second phase, more extensive measurements of EMF levels were made in the home for all children indicated by phase I to be in the top 10% of exposures, together with the corresponding matched case or control. The value taken to approximate the 90th percentile of the phase I measurement was 0.1 μT. Also included in phase II were individuals living in the proximity of high voltage overhead lines and underground cables and those with appliances specified in the phase I questionnaire.

Measurements were made after diagnosis, because of the retrospective nature of the study. Retrospective line load data for relevant high voltage lines were obtained from the electricity industry for the time when measurements were taken and for the year of interest, i.e. the year before diagnosis or for controls pseudo-diagnosis. The line load data were used to compute fields for both the year of interest and for the measurement period. These data were used to adjust the measurements to generate a historical exposure estimate for the year of interest.

To prevent identification of high and low measurements by study technicians, meter readings were not displayed. Information on the levels of EMFs in individual homes and schools were provided to study participants on request, but otherwise remained strictly confidential.

### The power of a two-phase approach to exposure measurement

It is straightforward to evaluate the power of a two-phase design compared either to a design in which all individuals have only phase I measurements, or to one in which all individuals have phase II measurements.

In the simple case where in the whole population the top p% of phase II exposures have a relative risk of $r$ compared to the remainder, and the phase I measurement is used to identify q% of individuals, there are approximately 2q% of case–control pairs on whom phase II measurements are made.

Figures 4A ($r = 1.5$) and 4B ($r = 2$) consider a study of 500 case–control pairs. The three curves give the power to detect a difference at the 5% significance level for a study in which all individuals have phase II measurements, a study in which all individuals have only phase I measurements, and the two-phase design as described. The benefit of the two-phase approach depends on the sensitivity and specificity of phase I compared to phase II, but for a wide range of values there is a major gain in power for relatively little cost, compared with a phase I only study.

### Inclusion

All families who had agreed to take part in the UKCCS were considered for inclusion in the EMF study. Eligibility for inclusion of both cases and controls was based on the eligibility of the home address, since exposure was based on household measurements.

Home addresses, of both cases and controls, were eligible if the child had lived there for 12 months or more before diagnosis or pseudo-diagnosis and was still living there. ‘Homes’ included fixed-site caravans, but not ‘mobile’ addresses. If a child was aged less than 12 months at (pseudo) diagnosis, an address was eligible if the child had lived there continuously between birth and the time of (pseudo) diagnosis. No attempt was made to measure previous homes.

If a case family was ineligible (did not live in current home for 12 months prior to diagnosis) or refused to take part in the EMF study, no control was invited to participate. Otherwise, the family of the matched control with the lower identification number was approached. If this first control family was ineligible or refused to participate, then the second control was approached and incorporated in Phase I, if eligible. If both controls were ineligible or refused to participate, the exposure of the case was not measured.

Due to delays in starting the EMF study, there was an interval of variable length between the date of (pseudo) diagnosis, and the date of the Phase I measurements. This affected the number of case–control pairs on whom measurements were available.

Schools were eligible if the child had attended them for 15 or more hours per week during the winter (October to March) immediately preceding (i.e. not including) the date of (pseudo) diagnosis. If the child attended more than one school in this period, the school in which the longest time was spent, in terms of the total number of hours, was chosen. ‘School’ included pre-schools in established, purpose-built buildings.

### Instrumentation

Commercially available Emdex II magnetic field meters (Enertech Consultants Ltd, USA) were used to measure resultant magnetic fields in the broadband frequency range 40–800 Hz. The meters incorporate three orthogonal sensing coils to measure true RMS magnetic flux density over the range 0.01–300 μT. The measurement resolution is 0.01 μT and the overall accuracy within the range 0.01–10 μT is ± 10% ± 0.005 μT. Computer memory inside the meter allowed data logging over extended periods. For short-

 *© 2000 Cancer Research Campaign*

term measurements, of two hours or less, magnetic flux density was recorded at sampling intervals of 1.5 and 3 s in the phase I and phase II assessments respectively. The sampling interval was adjusted to 10 s for longer measurements.

The instruments were calibrated in a Helmholtz coil facility which had an uncertainty of ±2.5%, traceable to national standards. Instruments were calibrated before issue to the study technicians and at intervals during the course of the study.

A check source generating a magnetic flux density of about 5 μT was developed to assess instrument integrity prior to and following each set of site measurements. General faults or the failure of any single sensing coil could be detected. Check source records were downloaded with measurement data following each site visit.

A subset of phase II case–control assessments also recorded electric field strength for a pilot study, using the same meters fitted with an external sensor. Based on laboratory tests the attenuation of magnetic flux density by the sensor was about 4%.

### Phase I residential measurements

The phase I residential measurements comprised in order: (i) three 3-min spot measurements taken in the centre of the child's bedroom, at the centre of the child's bed, and on the centre of the child's pillow; (ii) a 90-min measurement taken in the centre of the main family room; (iii) a repeat of the three spot measurements after the 90-min measurement.

Apart from the readings made on the bed, all these measurements were made with the instruments placed at a height of 1 m from the floor in a polypropylene stand and at least 1 m from any operating appliances.

The aim was to measure the homes of the case and of the corresponding control less than 4 months apart. If the period between measurements was greater than this, the earlier measurement was repeated. If a household measurement was repeated, the earlier reading was retained for study of repeated measurements.

During the phase I household visits, questions were asked about the time that the child spent in bed and at school, which were used in the calculation of time-weighted exposures.

### Phase II residential measurements

The matched case–control pair of phase II measurements were made as close as possible to each other and within a 4-week period.

Where the phase I questionnaire identified that a child's exposure might include exposure from a night storage heater or underfloor heating in the bedroom, then the phase II measurements covered a period during which the particular appliance was in use, typically during the winter months.

Phase II measurements took place during a period agreed with the appropriate electricity company as 'a period of typical operation of the local distribution system'. The measurements comprised: (i) four 3-min spot measurements taken at the centre of the family room, at the bedside position to be used for the 48-h measurement, at the centre of the child's bed and at the centre of the pillow; (ii) a 48-h measurement taken by the side of the middle of the child's bed; (iii) a repeat of the four spot measurements after the 48-h measurement.

Again, apart from the readings made on the child's bed, all of these measurements were made with the instruments placed at a height of 1 m from the floor in a polypropylene stand and at least 1 m from any operating appliances. Tamper-proof holders were used for the extended 48-h measurements by the side of the child's bed.

### Measurement in schools

School measurements took place when the heating systems were operating normally, which in England & Wales was taken to be the months October to March inclusive. There were two measurement schemes. The first was used when the child occupied a single classroom for the majority of the time spent at school during the relevant winter period, typically in primary schools. This scheme consisted of five 2-min spot measurements near the centre and four corners of this single room. When many classrooms were used, typically in secondary schools, spot measurements were made in up to five of the rooms most frequently used during the relevant winter period. In each of these rooms, a single measurement was made near the room centre, the measurement time totalling 10 min and the measurements in the different rooms being of equal duration. All measurements were made at a height of 1 m from the floor and at least 1 m from any operating mains appliance.

### Appliances

During the phase I visit, questions were asked on use of underfloor heating and night-storage heating in the house including the child's bedroom, with observation of position and type. These appliances were chosen firstly on the basis that the position of the appliance and child were well defined over the period of use and secondly that the appliance field contribution over the likely period of use could increase mean exposure from less than 0.1 μT to greater than 0.2 μT.

Where such an appliance was identified and used, the case–control pair qualified for the phase II assessment; this was carried out when the night-storage heater/under-floor heating was operating normally. Using appropriate periods of the 48-h measurement, specific exposure calculations were undertaken for these appliances, their contribution considered to be material only during the winter night-time. Use of an electric blanket was also ascertained; if a blanket was used and was available, permission was asked to send it to NRPB for detailed magnetic flux density measurements to assess the exposure.

In phase I exposure estimates, computed appliance field over the relevant period of use was combined with the measured background field by means of root sum of squares (RSS). Algorithms for computing appliance field were derived from investigations in the laboratories of NRPB and electrical testing houses, corroborated by evidence from UKCCS measurements. The same approach was adopted for phase II where there was no operating heating appliance during the overnight bedroom measurements.

Further questions were asked on other specific household sources of EMF (hairdryers, microwave ovens, electricity meters and central heating systems) to assess potential misclassification. This is to be the subject of secondary analyses.

### External sources

An External Sources Questionnaire (ESQ) was completed for each EMF study participant to identify important sources of electricity

*© 2000 Cancer Research Campaign*

supply close to homes and schools. Designed in cooperation with National Grid Company (NGC) and the Regional Electricity Companies (RECs) for England & Wales, and Scottish Power and Scottish Hydro Electric in Scotland, the specific purposes of the questionnaire were as follows: to identify high voltage lines and/or underground cables that had the potential to produce annual average field levels above 0.1 μT at the home and/or school; to obtain load and other circuit information to enable reconstruction of historical exposure; to check that the electricity distribution system was operating typically at the time of measurement; to identify substations and particular types of low voltage circuits that were near to the location of interest. The ESQs, blinded with respect to case–control status, were evaluated by NRPB.

Entry into phase II, on the basis of the ESQ was determined by the following criteria for England & Wales: (i) an NGC line within 400 m or underground cable located within 100 m of the home; (ii) a REC line of 66 kV or above, located within distances up to 200 m from a home; (iii) a REC line of 11–33 kV located within distances up to 80 m from a home; (iv) an operating substation or a phase-separated underground cable of 33 kV or above within 20 m; (v) a 3-phase 415 V distribution circuit located within 2 m of the home; (vi) atypical conditions of distribution circuits. In Scotland, equivalent criteria based on line voltages were used.

The threshold distances used to determine the relevant phase II circuits were based on design rating and therefore judged to be conservative. Typical loads on a REC line were found to be less than 20% of the circuit rating. Analysis of load data from a sample of NGC circuits during a winter period had indicated previously that 50% and 95% of the circuits had respective average loads of less than 30% and 50% of their rating (D Renew, NGC, personal communication).

External sources questionnaires were issued also for the non-measured participants in the main study. These were interviewed cases and controls who were either ineligible for EMF measurements (because they had moved house either during or since the time of interest) or who were eligible but had declined to participate in this part of the study. To investigate the possible effects of refusal bias, ESQs were also issued for a random sample of one thousand (approximately two-thirds) of the first-choice controls who had refused to take part in the full study.

### Historical exposure computations

Line load data were requested for all overhead lines whose voltages were 66 kV and above within certain threshold distances from the location of interest. For REC lines, threshold distances were used to identify homes and schools where the annual average magnetic field could exceed 0.15 μT. The distances were based on maximum loads as defined by the circuit rating, and relative phasing information. In practice, loads were well below the circuit rating as described in the previous section. For transmission lines, the annual load current or half of the average circuit rating and other detailed operational information, were used to define line-specific threshold distances. These distances were established for annual average magnetic fields of 0.1 μT. On a similar basis, load data were also requested for phase-separated underground cables of 66 kV and above, located within 20 m of the property.

National Grid Company's EM2D program was used to compute magnetic field levels. The program, evaluated by NRPB for the purposes of the study, was used to calculate the RMS magnetic flux density averaged over the measurement period and over the year of interest, which were used in the exposure algorithm. To set up a model, circuit parameters including line/tower type, conductor height above ground, distance, measurement height, phasing of circuits and current direction, were used as input data. A three-dimensional (3D) version of the program was used in some circumstances.

Load data contemporaneous with the period of measurement and the year of interest were requested. These were usually provided as a continuous series of load current/power values for each circuit, sampled at 1-min intervals for phase I, and at half-hour intervals for the year of interest. To allow for the temporal characteristics of line loading (Kaune et al, 1998), the values were sorted into separate date–time files to cover the appropriate periods of the year of interest. Where companies could not provide data for the relevant period, data for the closest alternative period were requested. If load data were not available, engineers were requested to provide best estimates based on available company records.

In a previous study carried out by NGC (Swanson, 1995), good agreement was found between instantaneous measured magnetic fields and the computed values. Using a similar computational approach to that used in the UKCCS, the error was estimated to be ± 6% at 0.1 μT. in the UKCCS, the typical overall uncertainty was estimated to be ± 20%, and checks with measurements implied worse case uncertainty of approximately ± 40% within 50 m of a power line, falling to ± 30% at 100 m. The additional computational error was mainly due to uncertain ESQ information, particularly the location and elevation data, and departures from the 2D modelling assumption.

In the UKCCS the year of interest exposure adjustment was made for all cases and controls where homes and schools attended met the criteria outlined above and for which load data were received. The calculation was based on a root sum/difference of squares method. A residual component, representing a constant 'local source' was derived by subtracting the contemporaneous calculated field from the measurement, and this RSS was combined with the year of interest calculated field. There were instances where the calculated field was greater than the measured field and these were ascribed to the inaccuracies in the parameters for the computational model, which have been described above. A comprehensive review was carried out of the modelling parameters used. Where calculated fields remained greater than the contemporaneous measured fields, the year of interest exposure was determined by adjusting the measured field by the ratio of the calculated fields for the two periods.

### Algorithm for combining measurements

On the basis of heating appliances contributing to exposure only during winter months, average exposure in the year preceding (pseudo) diagnosis was estimated using the following algorithm:

$$\text{Average exposure} = W_1.(\text{Bed: Winter Exposure}) + W_2.(\text{Bed: Summer Exposure}) \text{ in year of interest} + W_3.(\text{School Exposure}) + W_4.(\text{Home Non-Bed Exposure})$$

The weights ($W_1$–$W_4$, $\Sigma W_i = 1$) were individually calculated for each child to reflect the time spent in bed and in school, as recorded in the study questionnaire. Where any of the information needed to compute the weights was missing, average, age-related values were used.

 *© 2000 Cancer Research Campaign*

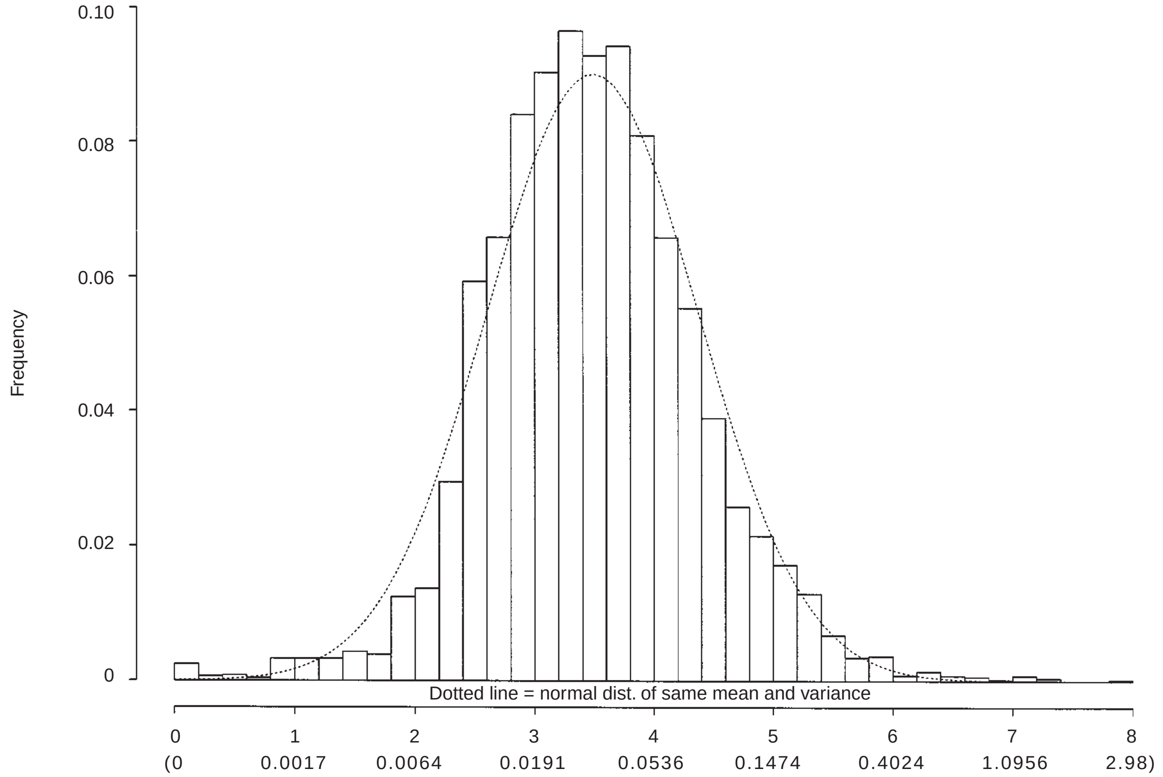

**Figure 5** Ln(1000*Average Total Exposure + 1), value retransformed to μT shown on second line. Mean = 0.0313, S.D. = 0.0313 (mean and var. of data as plotted, retransformed to microtesias), *n* = 4969

**Table 7** Correlation between different elements of phase I and phase II, for log-transformed data (*n* = 1185)

| | Phase I | | | Phase II | | |
|---|---|---|---|---|---|---|
| | **AREPI** | **BedPI** | **Non-bedPI** | **ETPII** | **BedPII** | **Non-bedPII** |
| AREPI | 1 | | | | | |
| BedPI | 0.95 | 1 | | | | |
| Non-bedPI | 0.87 | 0.73 | 1 | | | |
| ETPII | 0.75 | 0.73 | 0.74 | 1 | | |
| BedPII | 0.75 | 0.75 | 0.70 | 0.89 | 1 | |
| Non-bedPII | 0.72 | 0.64 | 0.80 | 0.82 | 0.81 | 1 |

AREPI: Phase I estimate of average residential exposure (= BedPI and Non-bedPI weighted by questionnaire data). BedPI: Average of the six spot measurements (two each in bed centre, pillow, bedroom centre) made in the bedroom during phase I. Non-bedPI: 90-min measurement made in family room during phase I. ETPII: 48-h extended time measurement made beside child's bed in phase II. BedPII: Average of the six spot measurements (two each in bed centre, pillow, 48-h measurement position) made in bedroom during phase II. Non-bedPII: Average of the two spot measurements made in family room during phase II.

In phase I, bed and home non-bed exposures were estimated from measurements in appropriate locations covered by a 2-h period. Bed exposure was estimated from bedroom spot measurements and the average of the 90-min family room measurement was used as an estimate of exposure for time not spent in bed or at school. In phase II, bed exposure and home non-bed exposure were estimated from the 48-h measurement and the phase I family room measurement. School exposure was common to both phase I and phase II.

Where necessary, to allow for changes in line loading and circuit configuration between the year of interest and the time of measurement, home and school measurements were adjusted to reflect the situation in the year before (pseudo) diagnosis. For bed-winter exposure, measurements were combined with appliance fields where appropriate. For bed-summer exposure, measurements were unadjusted.

### Characteristics of the phase I and phase II measures

The validity of the measures used in this study has been evaluated against the use of personal monitors in a substudy carried out by NRPB. This 'gold standard' validation study, estimated individual exposure by the use of personal monitors in three separate weeks during the year on a sample of 100 healthy children. It will be

© 2000 Cancer Research Campaign

**Table 8** Correlation between estimates of average residential exposure (AREPI) for repeat phase I measurements, for log-transformed data (median time between measurements = 1.41 years)

| Time between measurements | Correlation | Sample size |
|---|---|---|
| <6 months | 0.39 | 3 |
| 6<12 months | 0.58 | 68 |
| 12<18 months | 0.72 | 64 |
| 18<24 months | 0.59 | 50 |
| 24<30 months | 0.69 | 61 |
| 30<36 months | 0.80 | 11 |
| 36<42 months | 0.78 | 47 |
| 42<48 months | 0.96 | 3 |
| 48<54 months | 0.73 | 5 |

**Table 9** Correlation between phase I (AREPI) and phase II (ETPII) estimates of average residential exposure by time between measurements, for log-transformed data

| Time between measurements | Correlation | Sample size |
|---|---|---|
| All measurements | 0.75 | 1185 |
| ≤ 1 year | 0.76 | 607 |
| > 1 and < 2 years | 0.75 | 396 |
| ≥ 2 years | 0.66 | 182 |

**Table 10** Cross-tabulation of phase I (AREPI) and phase II (ETPII) estimates of average residential exposure with percentage figures in brackets (*n* = 1185)

| | ETPII < 0.1 μT | 0.1 μT ≤ ETPII < 0.2 μT | ETPII ≥ 0.2 μT |
|---|---|---|---|
| AREPI < 0.1 μT | 835 (70.5%) | 27 (2.3%) | 2 (0.2%) |
| 0.1 μT ≤ AREPI < 0.2 μT | 133 (11.2%) | 75 (6.3%) | 17 (1.4%) |
| AREPI ≥ 0.2 μT | 29 (2.4%) | 31 (2.6%) | 36 (3.0%) |

**Table 11** Number of eligible case–control pairs by diagnostic category

| Category | No. of pairs |
|---|---|
| Acute lymphoblastic leukaemia | 906 |
| Other leukaemias | 167 |
| Non-Hodgkin's lymphoma | 165 |
| Hodgkin's disease | 74 |
| Central nervous system tumours | 387 |
| Other malignancy | 527 |
| Total | 2226 |

reported elsewhere. The sample was chosen to have 50 children from 50 households with phase I measurements less than 0.1 μT, and 50 with phase I measurements greater than 0.1 μT.

### *Phase I measurements*

The distribution of phase I measurements based on nearly 5000 measurements to the end of the study is displayed for annual average exposure, on a log scale, in Figure 5. The distribution is strikingly close to log normal, with a geometric mean of 0.032 μT, and a retransformed standard deviation of 0.031 μT. The mean phase I exposure level falls between the geometric mean levels of 0.029 and 0.037 μT, published previously for UK homes (Merchant et al, 1994*a*, 1994*b*; Preece et al, 1996; Swanson and Kaune, 1999). The residential and school components of the total exposure have a similar distribution. There was some evidence of diurnal and seasonal variation in phase I measurements, reflecting the known daily and seasonal changes in the use of electricity.

Table 7 indicates the correlation between the different components of the phase I measurements, and with the overall residential exposure from phase I. All correlations are given for data on a log scale.

Table 8 gives the correlation between phase I measurements taken at different times in the same home, by the time interval between the two measurements. The data include a sample taken in Scotland to examine replication of phase I measurements. The correlation shows no decrease with time, for intervals of up to 4 years between measurements.

These phase I Tables, and those for phase II, are based on all measurements taken. This includes measurements on individuals who will not be included in the final analysis; for example, those for whom the matching case or control was not measured, or those found to be ineligible for the study as a whole.

### *Phase II measurements*

By the end of the study, nearly 1200 phase II measurements were made. Seasonal variation similar to that in the phase I data was observed.

Table 7 gives the correlation between the different component measures in phase II and with an overall phase II household measure based on the 48-h bedside measurement, together with the corresponding phase I measures, all on a logarithmic scale. Table 9 gives the correlations between phase I and phase II estimates of total residential exposure, for different intervals of time separating them. The correlation is only weakly related to the length of the interval. The correlations are higher in Table 9 than in Table 8, which reflects the greater variance among individuals on whom phase II measurements are taken. The standard deviation of the differences between the two measurements (on a logarithmic scale) is almost identical in the two situations, about 0.8.

The relationship between the two measurements can also be expressed in terms of sensitivity and specificity as mentioned earlier. Table 10 cross-tabulates the two measures in terms of the cut-off for phase II, i.e. 0.1 μT, and the level at which risk is hypothesized to be elevated, i.e. 0.2 μT. The individuals in Table 10 with phase I measurements less than 0.1 μT represent only 15% of all such individuals, whereas all those with phase I measurements greater than 0.1 μT should have phase II measurements and thus be included in this Table. With this correction, the sensitivity and specificity of phase I with respect to phase II are 0.80 and 0.98 respectively. As can be seen from Figure 4A and B, these values suggest that the two-phase approach will lead to a substantial increase in power and be close to that achieved by taking phase II measurements on everyone.

### Analysis

The diagnostic categories on which the primary analysis will focus are defined by the full study hypotheses, namely all leukaemias, ALL, CNS tumours and others.

The data relating to EMF exposure are classified in three groups.

In Group A, complete data (i.e. residential and school measurements where necessary and external source data) are available for both members of each case–control pair. It includes approximately

 *© 2000 Cancer Research Campaign*

**Table 12** Summary of measurements made in control houses

| Category | Numbers | % |
|---|---|---|
| Households | | |
| Potential households[a] | 12 757 | 100.0 |
| Households which refused detectors | 5774 | 45.3 |
| Households with detectors sent | 6983 | 54.7 |
| Total measured households | 5678 | 44.5 |
| Detectors | | |
| Despatched | 13 966 | 100.0 |
| Total returned by household | 11 899 | 85.2 |
| No measurement given | 66 | 0.5 |
| Faulty | 164 | 1.2 |
| Incorrect dates of placement | 8 | < 0.1 |
| Total valid detectors | 11 661 | 83.5 |
| Not in place for 5–7 months | 144 | 1.0 |
| Only one room measured | 161 | 1.2 |
| Total detectors analysed | 11 356 | 81.3 |

[a]Households lived in for more than six months by the child.

60% of all interviewed cases and a corresponding number of controls, not all which were necessarily first choice.

In Group B, EMF measurements are not available for each member of a pair, but external source data are available both for the case and the first-choice control.

In Group C, external source data are available for a random sample of 1000 of the 1582 first-choice controls for the cases with measurements in Group A that did not have full measurements and may or may not have had family interviews.

The primary analyses have been based on the case–control pairs with full EMF data available (Group A). The numbers of such case–control pairs for each main diagnostic category are given in Table 11. Approximately 20% of case–control pairs had phase II measurements. Annual average exposures based on the phase II measurements were used in the analysis when available on both a case and the corresponding control. If a phase II measurement was available for only one of a case–control pair, then phase I measurements were used for both. For the remaining 80% of case–control pairs, the annual average exposure based on the phase I measurement was used.

We will use as the exposure of interest the arithmetic average exposure in the year preceding (pseudo) diagnosis. Analyses will focus on comparisons between high (greater than 0.2 μT) and low (less than 0.1 μT) exposures and on a linear relationship between risk and dose.

### RADON AND TERRESTRIAL GAMMA RADIATION

Environmental exposure to low-level ionizing radiation is principally from radon and its decay products, cosmic rays, terrestrial gamma rays and radionuclides in food and drink (Hughes and O'Riordan, 1988). The ability of high linear-energy transfer alpha particles present in the terrestrial environment to cause cell damage, potentially leading to malignancy, has prompted particular interest. Radon (including its decay products) is established as a possible cause of lung cancer (BEIR IV, 1988) but other health effects have been suggested such as leukaemia in both adults and children (Henshaw et al, 1990).

Radon gas tends to become trapped within houses and may prove to be a source of a significant dose of alpha radiation. Representative measurements of the amount of radon in homes have been made on a national scale by the NRPB (Wrixon et al, 1988). Radon levels, however, vary between adjacent houses and direct measurements rather than geographic generalizations are essential to determine the levels to which individuals have been exposed. Attempts have, therefore, been made to measure the levels of radon in the present and past homes of all the children participating in the UKCCS.

Absorbed dose rates from external gamma sources are about twice as great indoors as outdoors in the UK. The mean indoor absorbed dose rate of 60 nGy per hour, which translates into an effective dose in dwellings of 280 $\mu Sv y^{-1}$, is lower than in most countries studied (United Nations Scientific Committee on the Effects of Atomic Radiation, 1993) but is based on survey data from some years ago and a sample size of 2300 households. External gamma radiation indoors has also been measured and this survey will materially add to knowledge of UK gamma distribution generally as well as specifically investigating any association with childhood cancers.

### Materials and methods

As part of the study, a face-to-face interview was conducted with each child's parents. A full residential history for the child was collected and all UK addresses lived in by the child, from birth to diagnosis, for 6 months or more were targeted for measurement of both radon gas and terrestrial gamma radiation. A written request to participate, in what was described as a 'Child Health Survey', was made to the occupants of each past residence, with a follow-up letter if there was no response to the first. Following agreement, detectors were sent to each household with instructions to place one in the main bedroom and one in the main living area. Two passive radon detectors, provided by the NRPB, were used to measure the concentration of radon gas (Wrixon et al, 1988) and two passive detectors were used to measure the terrestrial gamma radiation. After 6 months had elapsed, a letter was sent recalling the detectors, which were returned to the NRPB in pre-paid envelopes for processing and measurement of the cumulative radon exposure indicated by the alpha-particle tracks made by the radon decay products. We describe here the methods by which exposure to radon was assessed, using the data obtained for the control children to illustrate it. Details of the less complex assessment of exposure to terrestrial gamma radiation will be reported later with the results.

### Measurement of radon exposure

Table 12 shows the number of control households approached, the number that participated for which measurement was obtained and the fate of the detectors that were sent out. Although detectors were intended to be in place for 6 months, the period over which measurements were actually made varied. The analyses were restricted to those measurements from detectors which had been in place for between 5 and 7 months within a household for both living room and bedroom. After excluding faulty detectors and other missing information, there were 11 356 detector measurements available for 5678 control households (44.5% of the initial total).

#### *Outdoor correction*

The modelling of seasonal correction factors assumes the frequency distribution of radon concentrations in households is log-normal. Previous research, based on the NRPB data, suggested

© 2000 Cancer Research Campaign the overall distribution followed a log-normal distribution more closely when each household measurement had 4 Bq m$^{-3}$ subtracted (Gunby et al, 1993). This was informally termed an 'outdoor correction' factor as limited surveys of radon indicated outdoor levels to be low, around 4 Bq m$^{-3}$. The assumption driving the 'outdoor correction' was tested on the UKCCS data prior to correction for season.

### *Seasonality*

The aim of the radon survey was to measure average concentration of radon gas over a year. Radon becomes trapped within a building, as it is drawn up from the underlying rock and released gradually from the house through windows, doors and other ventilation. Different levels of heating and ventilation throughout the course of a year cause radon levels to fluctuate by season (Pinel et al, 1995). Due to the constraints of time placed on an epidemiological study, it is usual for radon detectors to be in place for approximately 6 months, therefore, a multiplicative factor is required to correct for seasonal variation. The NRPB have derived corrections from their own national survey of houses (Wrixon et al, 1988). These, however, are not based on the same sampling frame as the UKCCS data which is for houses in which there are young children. For this reason, seasonal correction factors were derived from the UKCCS sample, extending standard methodology by testing the validity of regional-specific factors. In broad terms, the methodology of Pinel et al (1995) uses the set of household measurements to calculate the mean concentration of radon for each month of the year; these monthly values are then used to derive seasonal correction factors.

The measurement, $m_i$ for each household $i$, obtained by the NRPB was in kilobequerels per cubic-metre hours (integrated exposure). This converts to a mean concentration of

$$M_i = \frac{m_i * 1000}{24 * p_i} \text{ in Bq m}^{-3}$$

where $p_i$ is the number of days the instruments were in place. Measurements often differ substantially between living room and bedroom, due to living rooms generally being closer to the ground floor; the source of radon. The NRPB surveyed times spent within different parts of the house (Wrixon et al, 1988) which suggested an average person spent 55% of their time in home in the bedroom and 45% in the other living areas. Previous UK studies (Darby et al, 1998) have used this division to allocate weighted average concentrations for each house, and this was used to calculate $m_i$ in preference to a simple average.

The mean concentration of radon for each month was calculated as

$$x_j = \frac{1}{N_j} \sum_{i\,T_j} \log(M_i)$$

for all months, $j = 1, \ldots, 12$ with the middle day of the measurement period for dwelling $i$ falling between the middle of the month $j-1$ and the middle of the month $j$. This ensures the measurement duration is symmetrical around the month it is assigned. $N_j$ is the number of measurements in the $j$th month. The most appropriate locational measure for a log-normal distribution is the geometric mean, which was used throughout the modelling procedures to estimate the average radon concentration. The geometric mean concentration for each month was calculated as $d_j = \exp(x_j)$.

A sine–cosine curve was fitted to the geometric mean monthly concentrations. The derived seasonal variation allows a correction factor to be calculation (Pinel et al, 1995).

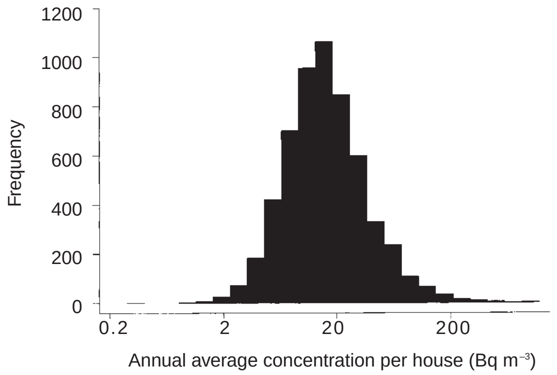

**Figure 6** Frequency distribution of annual average radon concentration in houses of controls (regionally adjusted for season)

### *Seasonality correction by region*

Seasonality is partly a function of the magnitude of variation in local climatic conditions, and the severity and lengths of seasons differ substantially across Great Britain. Therefore, it seems reasonable that seasonality in levels of radon concentration may vary across the UKCCS study regions, a hypothesis tested formally using these data. Mean monthly radon concentrations were calculated separately for households within each UKCCS study region, and these estimates were used with an indicator variable for the study region as a formal test of whether estimated coefficients were significantly different between regions. Statistically significant differences would support use of region-specific seasonal correction factors.

### Results

The frequency distribution of the radon concentration by household (regionally adjusted for season) is log-normally distributed, as would be expected for data of this type (Figure 6). Prior to seasonal correction the annual average radon concentration (arithmetic mean) for all households was 21.32 Bq m$^{-3}$, ranging between 0.36 and 1192.97 Bq m$^{-3}$ (Table 13). Regional annual averages, prior to seasonal correction, ranged from 16.36 Bq m$^{-3}$ in Scotland to 34.43 Bq m$^{-3}$ in the South West study region. Household measurements of radon concentration, prior to 'outdoor correction', closely follow a log-normal distribution and there was no improvement in fit after the correction (data not shown).

Seasonal correction factors for a detector in place for 6 months exactly are shown in Table 14. The measure of the proportion of variation in the data explained by the model $R^2$, are reported. All models fit the data with between 0.50 (South West) and 0.79 (Trent) of the variation explained by the seasonal curve. The model which used mean monthly radon concentrations calculated for each region separately, with an indicator variable for region, gave a significantly better fit than a model without the term

*© 2000 Cancer Research Campaign*

**Table 13** Annual average concentrations of radon in control houses in each study region (not seasonally adjusted)

| | | Annual household radon concentration (Bq $m^{-3}$) | | | | | |
|---|---|---|---|---|---|---|---|
| **UKCCS study region** | ***n*** | **Arith mean[a]** | **s.d.** | **Geo mean[b]** | **s.d.[c]** | **Minimum** | **Maximum** |
| Central/South Wales[d] | 538 | 26.96 | 39.60 | 18.84 | 2.12 | 2.22 | 542.08 |
| East Anglia | 467 | 21.29 | 15.94 | 17.16 | 1.92 | 2.38 | 118.89 |
| North East | 745 | 23.62 | 31.65 | 16.19 | 2.21 | 2.34 | 340.19 |
| North West | 772 | 17.72 | 26.74 | 12.12 | 2.15 | 1.32 | 354.48 |
| Scotland | 860 | 16.36 | 36.09 | 10.95 | 2.19 | 0.36 | 886.70 |
| South East | 429 | 22.25 | 20.49 | 16.22 | 2.21 | 1.84 | 165.34 |
| South Midlands | 573 | 30.52 | 63.85 | 18.97 | 2.36 | 1.19 | 1192.97 |
| South West | 731 | 34.43 | 66.36 | 19.70 | 2.60 | 0.91 | 1179.20 |
| Trent | 563 | 28.64 | 29.48 | 20.40 | 2.20 | 1.93 | 234.68 |
| All regions | 5678 | 24.32 | 30.42 | 15.95 | 2.30 | 0.36 | 1192.97 |

[a]Arithmetic mean. [b]Geometric mean. [c]Exponential of the geometric standard deviation. [d]The South Wales Study region was combined with Central.

**Table 14** Seasonal correction factors and model fit for a 6-month placement of the detector derived from control houses

| | | Correction factor for month of placement | | | | | | | | | | | |
|---|---|---|---|---|---|---|---|---|---|---|---|---|---|
| **UKCCS study region** | **$R^2$** | **Jan** | **Feb** | **Mar** | **Apr** | **May** | **Jun** | **Jul** | **Aug** | **Sep** | **Oct** | **Nov** | **Dec** |
| Central/South Wales | 0.77 | 1.15 | 1.19 | 1.16 | 1.09 | 1.00 | 0.93 | 0.88 | 0.86 | 0.88 | 0.92 | 1.00 | 1.08 |
| East Anglia | 0.55 | 1.01 | 1.08 | 1.14 | 1.16 | 1.13 | 1.07 | 0.99 | 0.93 | 0.89 | 0.88 | 0.90 | 0.94 |
| North East | 0.57 | 1.05 | 1.13 | 1.18 | 1.17 | 1.11 | 1.03 | 0.95 | 0.89 | 0.87 | 0.87 | 0.91 | 0.97 |
| North West | 0.72 | 1.07 | 1.12 | 1.14 | 1.12 | 1.06 | 1.00 | 0.94 | 0.90 | 0.89 | 0.91 | 0.94 | 1.00 |
| Scotland | 0.50 | 1.04 | 1.13 | 1.19 | 1.19 | 1.14 | 1.05 | 0.96 | 0.90 | 0.86 | 0.86 | 0.89 | 0.96 |
| South East | 0.55 | 1.20 | 1.32 | 1.34 | 1.24 | 1.09 | 0.95 | 0.86 | 0.80 | 0.80 | 0.84 | 0.92 | 1.05 |
| South Midlands | 0.53 | 0.96 | 1.04 | 1.12 | 1.17 | 1.18 | 1.13 | 1.05 | 0.97 | 0.90 | 0.87 | 0.87 | 0.90 |
| South West | 0.50 | 0.98 | 1.01 | 1.03 | 1.04 | 1.05 | 1.04 | 1.02 | 0.99 | 0.97 | 0.96 | 0.96 | 0.97 |
| Trent | 0.79 | 1.51 | 1.43 | 1.23 | 1.02 | 0.87 | 0.78 | 0.75 | 0.77 | 0.84 | 0.98 | 1.18 | 1.40 |
| All regions | 0.51 | 1.04 | 1.08 | 1.09 | 1.08 | 1.05 | 1.01 | 0.97 | 0.93 | 0.92 | 0.93 | 0.95 | 0.99 |

(Table 15). This provides confirmation that the calculation of seasonality correction factors on a region-specific basis is appropriate.

Table 16 shows the annual average household concentration of radon for each study region after applying the region-specific seasonality correction factors. The South West study region continued to measure the highest average levels, 34.47 Bq $m^{-3}$ and Scotland the lowest, 16.74 Bq $m^{-3}$. Table 17 divides the households into categories, the highest category containing 0.7% of all measured control households, being that equal to or above the 'action level' of 200 Bq $m^{-3}$, determined by the NRPB as the level above which remedial work to a house should be carried out.

**Table 15** Model fit and the effect of region on the seasonal model for controls

| Regions | Coefficient (95% CI)[a] |
|---|---|
| North West (baseline) | 0 (–) |
| North East | **6.22 (2.54–9.89)** |
| South West | **8.06 (4.39–11.73)** |
| Trent | **7.69 (4.02–11.36)** |
| Central and South Wales | **6.89 (3.22–10.56)** |
| East Anglia | **6.33 (2.66–10.00)** |
| South Midlands | **6.96 (3.29–10.63)** |
| South East | **5.36 (1.69–9.03)** |
| Scotland | 0.64 (–3.03–4.31) |
| Likelihood ratio $\chi^2$ (*P*-value)[b] | **39.55 (< 0.001)** |
| R[a] | 0.38 |

[a]Significant at $P < 0.05$ in bold. [b]Test against the seasonal model only. [c]Compared with a value of 0.11 without the regional correction

### Discussion

The UKCCS has collected radon gas measurements for a large number of residences, which are linked to information about the families living there. These measurements will provide the basis for an epidemiological analysis of the association between radon in homes and the incidence of childhood cancer. The study for which these measurements were made involved a population-based sample of children with cancer and two controls. The homes for the selected control children were representative of homes in the general population containing children of the same age and sex as the affected children. Satisfactory measurements were, however, obtained for less than half the target number and the effect of non-compliance on the risk estimates will have to be taken into account in studies of the association of childhood cancer with radon exposure.

The levels of radon gas in homes were generally low, with 74% of households having annual average radon concentrations of less than 25 Bq $m^{-3}$. The seasonally adjusted (arithmetic) average annual concentration for the entire study, 24.7 Bq $m^{-3}$, was higher than the NRPB estimates for UK housing of 20.5 Bq $m^{-3}$ (Wrixon et al, 1988). This may reflect the differences between the sampling frames, the present one being houses with children, and between the UK housing stock at the two periods. The low proportion of households exceeding the government 'Action Level', 0.7% above 200 Bq $m^{-3}$, is consistent with, but higher than, the national average of 0.5% (Kendall et al, 1988).

© 2000 Cancer Research Campaign 

**Table 16** Annual average concentrations of radon in control houses in each study region (seasonally adjusted by region)

| | | Annual household radon concentration (Bq m$^{-3}$) | | | | |
|---|---|---|---|---|---|---|
| **UKCCS study region** | **Number** | **Arith mean[a]** | **s.d.** | **Geo mean[b]** | **s.d.[c]** | **Minimum** |
| Central/South Wales | 538 | 27.70 | 40.74 | 19.38 | 2.12 | 2.22 |
| East Anglia | 467 | 22.18 | 16.62 | 17.82 | 1.93 | 2.54 |
| North East | 745 | 24.40 | 31.76 | 16.81 | 2.21 | 2.59 |
| North West | 772 | 17.88 | 27.46 | 12.20 | 2.15 | 1.24 |
| Scotland | 860 | 16.74 | 32.36 | 11.49 | 2.17 | 0.41 |
| South East | 429 | 22.80 | 20.28 | 16.79 | 2.19 | 2.21 |
| South Midlands | 573 | 29.81 | 58.01 | 18.86 | 2.35 | 1.06 |
| South West | 731 | 34.47 | 66.40 | 19.73 | 2.61 | 0.91 |
| Trent | 563 | 29.33 | 32.98 | 20.58 | 2.22 | 2.04 |
| All regions | 5678 | 24.69 | 40.65 | 16.30 | 2.29 | 0.41 |

[a]Arithmetic mean. [b]Geometric mean. [c]Exponential of the geometric standard deviation.

**Table 17** Numbers of control houses in pre-defined categories of annual average radon concentration (seasonally adjusted by region)

| Average annual radon concentration (Bq m$^{-3}$) | *n* | % |
|---|---|---|
| 0–24 | 4173 | 73.5 |
| 25–49 | 1000 | 17.6 |
| 50–99 | 355 | 6.3 |
| 100–199 | 110 | 1.9 |
| 200 and over (‘action level’) | 40 | 0.7 |
| Total | 5678 | 100.0 |

In contrast to our findings, previous research based on NRPB data suggested that the overall distribution of radon followed a log-normal distribution more closely when the entire data set had 4 Bq m$^{-3}$ removed (Gunby et al, 1993). This was informally termed an ‘outdoor correction’ factor as limited surveys of radon indicated outdoor levels to be low: around 4 Bq m$^{-3}$. Unfortunately, exact comparisons with our data could not be made as the results presented by Gunby et al (1993) were truncated, and omitted the first 5% of the data. The occupancy patterns used for the allocation of a weighted average household radon level were from a national study based on persons of all ages. Two small studies carried out for the UKCCS on children do not contradict these findings.

The data were examined regionally because of concern that seasonal patterns in radon concentrations may vary across the country because of differences in climate, housing type and behaviour. Examination of the data confirmed the fact that seasonal correction factors would be more appropriately calculated on a region-specific basis. The study regions were defined a priori for the purposes of data collection, were geographically contiguous and contained approximately similar numbers of persons. To check that the hypothesis can be supported by the data, the sine–cosine seasonality curve was modelled for region-specific mean radon concentrations. The model fit was improved after adding a variable for the region, as would be expected after inspection of the seasonal correction factors, with Scotland and the North West being significantly different from the other study regions.

The data set described here documents an important profile of radon gas concentrations in a specific section of UK housing; namely, that inhabited by children. This is the largest data set of measurements aimed not at identifying areas affected by high levels of radon (Miles et al, 1996) but at characterizing a population-based sample of the population. The appropriate allowance for geographical differences in seasonality provides a basis for epidemiological investigations into the association between radon and childhood cancer.

### DIAGNOSTIC REVIEW AND CLASSIFICATION OF SOLID TUMOURS

For the purposes of data analysis cases must be classified according to diagnosis into a manageable number of groups. Descriptive epidemiological data on cancer are often presented in terms of categories in the neoplasms chapter of the International Classification of Diseases (WHO, 1992). This system is based on anatomical site and while this is broadly satisfactory for adult onset cancers, where over 85% of non-haematological malignancies are carcinomas (Berg, 1996) this is not satisfactory for paediatric cancers which form a specialized group of rare neoplasms with adult-type carcinomas being exceedingly uncommon (Stocker and Askin, 1998). Paediatric cancer data are more satisfactorily grouped, utilizing the International Classification of Diseases for Oncology (ICD-O) (WHO, 1990). In this system tumours are described in terms of morphology and topography. The second edition of ICD-O (ICD-O2) includes more than 1600 morphology codes (M codes) and more than 300 topography codes (T codes), consequently hundreds of thousands of M and T code combinations are possible. A system that groups combinations of M and T codes together is, therefore, required to provide a smaller number of diagnostic categories for practical purposes. This report describes the diagnostic review of non-leukaemia cases and the classification scheme based on ICD-O2 which has been employed to group cases for analysing data to test the UKCCS hypotheses.

### The review process

All but the leukaemia cases were eligible for diagnostic review regardless of whether the children’s parents were subsequently interviewed for the study. Registration details were obtained principally from the UKCCSG Data Centre. During the period of the study more than 80% of all incident cases of cancer in children in England, Scotland and Wales were referred for initial treatment to UKCCSG paediatric oncology member centres. Registration details for remaining cases were obtained from the UKCCS regional epidemiology centres or, for a small number of cases, through the National Register of Childhood Cancers. UKCCS registration numbers for each eligible case were obtained from the

 *© 2000 Cancer Research Campaign*

**Table 18** Interviewed cases by diagnostic group

| | Age/Sex | | | | | | | | | |
|---|---|---|---|---|---|---|---|---|---|---|
| | 0 | | 1–4 | | 5–9 | | 10–14 | | 0–14 | |
| **Diagnostic group** | **M** | **F** | **M** | **F** | **M** | **F** | **M** | **F** | **M** | **F** |
| Unclassified | 0 | 0 | 0 | 0 | 0 | 1 | 1 | 0 | 1 | 1 |
| Hodgkin's disease | 0 | 0 | 8 | 1 | 26 | 9 | 50 | 24 | 84 | 34 |
| Non-Hodgkin's lymphoma | 0 | 0 | 32 | 20 | 55 | 18 | 81 | 25 | 168 | 63 |
| Unspecified lympho-reticular neoplasms | 0 | 0 | 1 | 0 | 3 | 0 | 1 | 0 | 5 | 0 |
| Total lymphoma and other lympho-reticular neoplasms | 0 | 0 | 41 | 21 | 84 | 27 | 132 | 49 | 257 | 97 |
| Pilocytic astrocytoma | 3 | 5 | 21 | 34 | 34 | 37 | 17 | 19 | 75 | 95 |
| Other low-grade astrocytomas | 5 | 1 | 9 | 13 | 7 | 15 | 17 | 17 | 38 | 46 |
| High-grade astrocytomas and glioblastomas | 1 | 1 | 4 | 7 | 7 | 14 | 7 | 7 | 19 | 29 |
| Other gliomas | 1 | 2 | 8 | 6 | 17 | 19 | 8 | 5 | 34 | 32 |
| Ependymoma | 2 | 2 | 20 | 11 | 7 | 8 | 10 | 9 | 39 | 30 |
| Choroid plexus tumours | 7 | 3 | 4 | 4 | 1 | 0 | 1 | 0 | 13 | 7 |
| Medulloblastoma | 4 | 4 | 22 | 17 | 34 | 25 | 18 | 5 | 78 | 51 |
| Other PNET | 3 | 2 | 7 | 10 | 1 | 5 | 3 | 2 | 14 | 19 |
| Other specified intracranial/intraspinal | 2 | 2 | 9 | 7 | 13 | 12 | 7 | 7 | 31 | 28 |
| Unspecified intracranial and intraspinal | 0 | 0 | 1 | 1 | 0 | 3 | 1 | 2 | 2 | 6 |
| Total CNS and misc intracranial and intraspinal | 28 | 22 | 105 | 110 | 121 | 138 | 89 | 73 | 343 | 343 |
| Retinoblastoma | 22 | 16 | 25 | 19 | 2 | 2 | 1 | 0 | 50 | 37 |
| Neuroblastoma and ganglioneuroblastoma | 42 | 21 | 51 | 42 | 16 | 9 | 5 | 2 | 114 | 74 |
| Peripheral neuroectodermal tumours – bone | 0 | 1 | 2 | 2 | 6 | 5 | 17 | 10 | 25 | 18 |
| Peripheral neuroectodermal tumours – extraskeletal | 1 | 2 | 4 | 2 | 5 | 6 | 6 | 9 | 16 | 19 |
| Total peripheral neural tumours | 43 | 24 | 57 | 46 | 27 | 20 | 28 | 21 | 155 | 111 |
| Wilms' tumour | 12 | 5 | 62 | 60 | 17 | 18 | 4 | 4 | 95 | 87 |
| Renal carcinoma | 0 | 0 | 0 | 0 | 0 | 0 | 2 | 3 | 2 | 3 |
| Other specified renal tumours | 3 | 1 | 2 | 2 | 4 | 0 | 0 | 0 | 9 | 3 |
| Total renal tumours | 15 | 6 | 64 | 62 | 21 | 18 | 6 | 7 | 106 | 93 |
| Hepatoblastoma | 5 | 5 | 11 | 4 | 0 | 1 | 2 | 0 | 18 | 10 |
| Hepatic carcinoma | 0 | 0 | 0 | 0 | 1 | 2 | 0 | 1 | 1 | 3 |
| Total hepatic tumours | 5 | 5 | 11 | 4 | 1 | 3 | 2 | 1 | 19 | 13 |
| Osteosarcoma | 0 | 0 | 0 | 3 | 11 | 8 | 17 | 17 | 28 | 28 |
| Chondrosarcoma | 0 | 0 | 0 | 0 | 0 | 0 | 1 | 1 | 1 | 1 |
| Embryonal rhabdomyosarcoma | 2 | 3 | 30 | 19 | 19 | 11 | 7 | 4 | 58 | 37 |
| Alveolar rhabdomyosarcoma | 1 | 1 | 7 | 4 | 1 | 3 | 2 | 3 | 11 | 11 |
| Other and unspecified rhabdomyosarcoma | 1 | 0 | 4 | 3 | 2 | 4 | 1 | 1 | 8 | 8 |
| Other specified bone tumours | 0 | 1 | 0 | 0 | 1 | 0 | 1 | 0 | 2 | 1 |
| Intracranial & intraspinal soft tissue tumours | 0 | 0 | 0 | 3 | 0 | 0 | 0 | 0 | 0 | 3 |
| Other specified soft tissue tumours | 3 | 4 | 6 | 1 | 10 | 6 | 11 | 9 | 30 | 20 |
| Unspecified soft tissue tumours | 1 | 1 | 4 | 1 | 3 | 1 | 4 | 0 | 12 | 3 |
| Total sarcomas and other mesenchymal tumours | 8 | 10 | 51 | 34 | 47 | 33 | 44 | 35 | 150 | 112 |
| Gonadal germ cell tumours | 4 | 0 | 14 | 2 | 0 | 5 | 5 | 5 | 23 | 12 |
| Intracranial & intraspinal germ cell tumours | 3 | 1 | 1 | 2 | 2 | 3 | 17 | 8 | 23 | 14 |
| Other non-gonadal germ cell tumours | 3 | 2 | 3 | 12 | 1 | 1 | 1 | 1 | 8 | 16 |
| Gonadal carcinoma | 0 | 0 | 0 | 0 | 1 | 0 | 0 | 1 | 1 | 1 |
| Total germ cell, trophoblastic and other gonadal tumours | 10 | 3 | 18 | 16 | 4 | 9 | 23 | 15 | 55 | 43 |
| Adrenal cortical carcinoma | 0 | 0 | 0 | 2 | 0 | 1 | 0 | 1 | 0 | 4 |
| Nasopharyngeal carcinoma | 0 | 0 | 0 | 0 | 1 | 0 | 3 | 0 | 4 | 0 |
| Thyroid carcinoma | 0 | 0 | 0 | 0 | 2 | 0 | 1 | 1 | 3 | 1 |
| Melanoma | 0 | 0 | 0 | 4 | 4 | 8 | 4 | 7 | 8 | 19 |
| Non-melanoma malignant neoplasms of skin | 0 | 0 | 0 | 0 | 0 | 0 | 1 | 4 | 1 | 4 |
| Other carcinoma NEC | 0 | 0 | 0 | 0 | 1 | 1 | 3 | 4 | 4 | 5 |
| Pancreatoblastoma and (pleuro) pulmonary blastoma | 0 | 0 | 1 | 2 | 0 | 0 | 0 | 0 | 1 | 2 |
| Other specified rare tumours | 0 | 0 | 0 | 0 | 0 | 1 | 0 | 0 | 0 | 1 |
| Total rare miscellaneous neoplasms | 0 | 0 | 1 | 8 | 8 | 11 | 12 | 17 | 21 | 36 |
| Unspecified malignant neoplasms NE | 1 | 2 | 0 | 1 | 0 | 3 | 0 | 1 | 1 | 7 |
| Langerhan's cell histiocytosis | 6 | 2 | 11 | 9 | 9 | 7 | 3 | 2 | 29 | 20 |
| Total all cases | 138 | 90 | 384 | 330 | 324 | 274 | 343 | 221 | 1189 | 915 |

respective UKCCS regional epidemiology centres to allow diagnostic data to be linked to the epidemiology data.

Pathology reports, and as appropriate, radiology reports were obtained for all non-UKCCSG cases and for those UKCCSG cases where this information was not appended to the registration form. More than half of all solid tumour cases were entered into national or international clinical trials. Special histopathological review is automatically carried out for all such cases entered into trials and it was agreed nationally that diagnostic review pathology reports for them would be made available to the UKCCS. Members of the pathology review panels associated with each clinical trial additionally agreed to review diagnoses of respective non-trial cases eligible for the UKCCS. Collation of clinical trial diagnostic review reports and organization and coordination of review of non-trial cases nationally was carried out centrally for the UKCCS at the CRC Paediatric & Familial Cancer Research Group

© 2000 Cancer Research Campaign

(PFCRG) in Manchester. Pathology review panels for Hodgkin's disease, non-Hodgkin's lymphoma, brain tumours, neuroblastoma, renal tumours, liver tumours, bone tumours, soft tissue sarcoma and germ cell tumours were already set up in association with clinical trials. An additional pathology panel was set up to review diagnoses of the remaining rare tumour groups. Thus, the diagnoses of all cases both clinical trial and non-clinical trial were reviewed by the same specialist panels.

Following receipt of registration details, confirmation of eligibility for UKCCS and ascertainment of a UKCCS registration number, clinical trial status was obtained from the UKCCSG Data Centre. For non-trial cases, histopathology material was requested by the coordinating centre in Manchester from the relevant consultant pathologists. Tissue sections were cut and mounted, and distributed to the appropriate diagnostic panel in accordance with the panel's requirements. Consensus reports were prepared on each case by the review panels and reports sent to the Manchester centre in due course. For trial cases a similar review process was followed but this was coordinated by the UKCCSG Data Centre in Leicester. For these latter cases, copies of the consensus pathology panel reports were provided to the coordinating centre in Manchester. These reports constitute the final reviewed diagnosis on each case.

Diagnoses were coded according to ICD-O2 and entered together with identification and other registration details onto the histopathology database held in Manchester. A hierarchical confirmation of diagnosis code was also allocated as follows:

1. special histopathological review (trial and non-trial)
2. review at UKCCSG member centre or other specialist oncological or neuropathology centre and pathology report received in Manchester
3. other pathology report received in Manchester
4. diagnosis histologically confirmed but pathology report not seen in Manchester
5. no histological confirmation but positive biological marker
6. clinical diagnosis with radiology report seen in Manchester
7. clinical diagnosis radiology report not seen in Manchester,
8. UKCCS registration form or notification from CCRG in Oxford with no accompanying pathology or radiology report.

Each case was allocated to a main diagnostic group and subgroup within the main group according to the classification scheme described below.

### Diagnostic classification scheme for childhood cancers

In formulating the classification scheme priority was given to allocating individual tumour types to diagnostic groups on the basis of what is currently known about the histogenesis and biology of the tumours. The following features were incorporated into the scheme: (i) the more common types of childhood malignancies were specified as individual categories, (ii) rare tumours of particular biological interest were also individually specified, (iii) diagnostic categories were organized in a hierarchical way to allow major groups to be sub-divided into sub-groups and where appropriate into sub-categories within sub-groups. This allowed flexibility with regard to the diagnostic specificity with which data may be analysed and presented while retaining a standard framework.

For each morphological entity, or group of entities defined by ICD-O M codes, a set of corresponding topography codes was also defined. A computer data input routine was developed such that only compatible paired M and T codes would be accepted and incompatible M and T codes would be unclassified. This arrangement acts as an automatic check at the data input stage on the accuracy of information received on morphology and primary site of tumours and the accuracy of coding. Furthermore, by these means common data input errors such as reversal of two digits would be detected at the outset. The draft scheme was tested on the Manchester Children's Tumour Registry database which was already coded in ICD-O2. Primary information on those cases that were not classified to a diagnostic group was checked and inconsistencies in the allocation of M and T codes within the draft scheme were corrected. The final scheme and accompanying data input programme were then used for the UKCCS diagnostic data.

The UKCCS case series includes some relatively recently described diagnostic entities for which ICD-O codes do not exist. Where it was thought that no appropriate alternative code existed, it was necessary to create a small number of new codes and to incorporate these into the classification scheme. The final scheme comprises 11 major diagnostic groups with a total of 45 subgroups. The distribution of interviewed cases included in UKCCS according to this classification scheme is shown in Table 18. Detailed results of the histopathology review will be presented elsewhere (Birch and Kelsey et al, in preparation).

### LEUKAEMIA: BIOLOGICAL SAMPLES AND CLASSIFICATION

### Rationale of classification

The acute leukaemias have been traditionally classified by morphological and cytochemical criteria into lymphoblastic (ALL) and myeloblastic (AML) sub-classes for diagnostic and treatment purposes (Henderson et al, 1996). Beginning in the 1970s, it became apparent that these two broad divisions disguised considerable biological heterogeneity. Subsequently, immunophenotypic and chromosomal variations (structural or numerical) were identified and standardized using monoclonal antibodies (Greaves et al, 1985; van Dongen and Adriaansen, 1996) and G-banded chromosome analysis (Pui et al, 1993; Raimondi, 1993) respectively. Over the past decade, changes in individual genes (rearrangements/fusions, mutations, deletions, etc) have been recognized and have provided a further hierarchical tier of leukaemia classification (Greaves, 1996*a*). The validity of this biological approach has been endorsed by the finding that subsets so defined had different prognostic outcomes in the context of particular therapies (Kersey, 1997; Pui and Evans, 1998). The design of current therapeutic trials or patient selection for bone marrow transplantation now exploits these biological classifications.

As acute leukaemia in children (and in adults) is not a single biological entity or disease, it seems highly improbable that its aetiology will be attributable to a single causal mechanism. Epidemiological case–control studies in the past have mostly divided cases into myeloid or lymphoid (the ICD system). Although both ALL and AML as well as CML may be initiated by ionizing radiation, there are several precedents in haematological malignancy for more selective or preferential aetiological associations. These include HTLV1 with mature T-cell leukaemia/lymphoma, EBV with Burkitt-like B-NHL, benzene with AML, certain genotoxic therapeutic agents with AML (Henderson et al, 1996; Smith et al, 1996; Greaves, 1997). The incidence rates or

 *© 2000 Cancer Research Campaign*

relative frequency of subtypes also appear to vary geographically, especially with respect to the age peak of incidence in ALL at 2–5 years of age which consists of B-cell precursor ALL or common c-ALL (Greaves et al, 1985, 1993). The prominence of height of this peak in different countries has changed asynchronously during the past 60 years (Greaves et al, 1985, 1993) and although changes in ascertainment or diagnosis may have contributed to this, c-ALL has been proposed as having a distinct infectious aetiology in which social circumstances modulate patterns of exposure (Greaves, 1997). c-ALL in turn is heterogeneous by molecular genetic criteria but two large subgroups predominate – hyperdiploidy and *TEL (ETV6)-AML1 (CBFA2)* gene fusion (Bernard et al, 1996; Secker-Walker, 1997).

Age is a prognostic factor in ALL and it is now recognized that the higher risk groups of ALL below 1 year of age or above 10 years seldom have the common molecular characteristics of c-ALL in the 2- to 5-year age peak. Infants (< 1 year) in particular appear to have a distinct biological subtype of disease in which translocations at 11q23 are predominant. These generate fusions of the *MLL* gene with partner genes on other chromosomes, especially *AF4* (at 4q) (Pui et al, 1995; Greaves, 1996*b*). Similar translocations involving 11q23/*MLL* (but with different partner genes) are common in paediatric AML, particularly below the age of 3 years. 11q23/*MLL* gene fusions are also frequent in secondary leukaemias arising from prior therapeutic exposure to drugs that inhibit the enzyme topoisomerase II. On this basis it has been proposed that transplacental exposure to chemically similar compounds in utero might provide a selective causal pathway for infant AML and ALL with 11q23/*MLL* rearrangements (Ross et al, 1994*b*; Greaves, 1997). Some prior epidemiological data suggest selective associations for acute leukaemia, and especially AML, in very young (< 2 years) patients (Ross et al, 1994*a*).

These data and insights all suggest that it would be prudent whenever possible to incorporate biological classification into epidemiological studies of leukaemia. This is only worthwhile, however, when studies are of a sufficient size to generate adequate numbers in the smaller subgroups.

This is the first large case–control study where a systematic biological classification has been undertaken. Of the five major hypotheses under discussion (see p. 1074), those involving EMF and ionizing radiation do not involve any prior consideration of biological subsets of ALL. With respect to chemical exposures (in utero) and infection, however, we have identified biological subgroups in advance for which selective associations will be sought (e.g. delayed infection and HLA associations in c-ALL).

As diagnostic samples of acute leukaemia were referred to a central reference laboratory (see below) for molecular genetic analysis, it was also decided to use whatever material remained after the tests were completed to establish a sample bank. This is designated for use in add-on studies related to our major hypothesis (e.g. screening for viruses or for gene polymorphisms by polymerase chain reaction (PCR) methods).

### Materials and methods

### *Morphology*

Individual haematologists in participating centres diagnosed ALL and AML based on morphological, standard staining (PAS ± Sudan Black ± non-specific esterase ± acid phosphatase stains) and immunophenotyping. Central review was performed by a panel of three haematologists as part of the MRC therapy trials protocol. This panel ascribed individual patients to an appropriate French–American–British morphological classification type; for ALL into L1, L2 or L3; for AML into M0–M7 (Catovsky et al, 1991; Lilleyman et al, 1992).

### *Immunophenotyping*

In 1989–1990, following national consensus meetings on the optimal battery of monoclonal antibodies required for appropriate immuno-phenotypic diagnosis of ALL, a series of workshops were held to produce consistent quality of performance in such testing, prior to the commencement of the MRC UKALL XI trial which ran throughout the period of the National Case Control Study. Thereafter centres admitting patients to the MRCs ALL trials were requested to perform a standard panel of immunophenotyping tests (Hann et al, 1998), which included anti-CD2, CD7, CD10, CD13, CD19, CD33, CD34 and HLA-DR as well as testing for terminal deoxynucleotidyl transferase (TdT) and for cytoplasmic immunoglobulin. The latter (cyto m) data have not been used for the classification scheme reported here.

In line with international agreement, a cut-off point for positivity was taken at ≥ 30% for CD2, CD7, CD19 and CD34. Patients were investigated by all 98 participating physicians using the same panel, but 42% of laboratories used flow cytometry analysis, 38% alternative methods in addition to flow cytometry and 20% used no flow cytometry and used only alternative immunophenotyping techniques (fluorescence microscopy, immunohistochemistry).

Cases of ALL were classified by immunophenotype into B-cell lineage or T-cell lineage subtypes. Cases that could not be unambiguously defined because of incomplete data or mixed lineage phenotypes were classified as ‘Other’. Seven per cent of eligible cases of ALL samples were not available for analysis.

Clinical presentation alone rarely provides clarity between the diagnosis of ALL or AML. The same battery of morphological staining and phenotyping was therefore performed on the majority of patients with AML, with the addition of erythroid and megakaryocyte antibody markers wherever appropriate. Central morphological review was also performed by the panel for AML cases.

### *Cytogenetics*

Conventional G-banded cytogenetic analysis was performed on diagnostic bone marrow and/or peripheral blood samples from ALL and AML patients by local cytogenetic laboratories. Cytogenetic data from patients entered to MRC treatment trials were routinely collected by two national databases: the Leukaemia Research Fund UK Cancer Cytogenetics Group ALL Karyotype Database (ALL Database) and the Kay Kendall Leukaemia Fund Childhood AML Karyotype Database (AML Database). Slides are reviewed before the karyotypes are entered to the ALL Database, and all karyotypes are written according to the International System for Human Cytogenetic Nomenclature (ISCN, 1995). Cytogenetic data were then provided directly from the ALL and AML Databases to the UKCCS.

### *Molecular diagnostics and cell/DNA/plasma banks*

Pretreatment biological samples from leukaemia and non-leukaemia cancer cases were referred to a single centre and entered into a FoxPro database. Of the 1711 leukaemia cases (ALL plus AML) entered into the study, biological samples were received from 1153. For the 2102 non-leukaemic cancers registered and

© 2000 Cancer Research Campaign

**Table 19** ALL and AML immunophenotypes

| | ALL | | | | | AML | Overall total |
|---|---|---|---|---|---|---|---|
| | B-cell | | T-cell | Other | Not done | | |
| | Common | Pro-B | | | | | |
| Sex | | | | | | | |
| Boys | 512 (56.7)[a] | 22 (45.8) | 90 (65.7) | 141 (51.8) | 55 (54.5) | 134 (53.6) | 954 (55.8) |
| Girls | 391 (43.3) | 26 (54.2) | 47 (34.3) | 131 (48.2) | 46 (45.5) | 116 (46.4) | 757 (44.2) |
| Age at diagnosis | | | | | | | |
| 0 | 20 (2.2) | 22 (45.8) | 1 (0.7) | 7 (2.6) | 0 (0) | 35 (14.0) | 85 (5.0) |
| 1 | 64 (7.1) | 3 (6.3) | 7 (5.1) | 15 (5.5) | 7 (6.9) | 36 (14.4) | 132 (7.7) |
| 2–5 | 570 (63.1) | 9 (18.7) | 50 (36.5) | 145 (53.3) | 53 (52.5) | 58 (23.2) | 885 (51.7) |
| 6–14 | 249 (27.6) | 14 (29.2) | 79 (57.7) | 105 (38.6) | 41 (40.6) | 121 (48.4) | 609 (35.6) |
| Down's syndrome | 17 (1.9) | 0 (0) | 0 (0) | 5 (1.8) | 2 (2.0) | 15 (6.0) | 39 (2.3) |
| Total | 903 (66.4)[b] | 48 (3.5)[b] | 137 (10.0)[b] | 272 (20.0)[b] | 101 | 250 | 1711 |

[a]Percentages of cases (in vertical columns). [b]Percentages of classified cases (in horizontal rows).

interviewed, a blood sample was received from 738. Leukaemic samples consisted of up to 5 ml of peripheral blood (with anti-coagulant) and/or up to five bone marrow smears. Blood only was received from non-leukaemic cancers.

On receipt of samples, slides were individually wrapped in foil and stored at – 70°C. Plasma was separated from blood by centrifugation, aliquoted and stored at –2°C or –70°C. Mononuclear cells were separated over a density gradient and counted. In cases with a low yield of cells from blood (< $10^6$) the cells were either pellet frozen in liquid nitrogen or stored in guanidinium isothiocyanate (GIT) solution for subsequent RNA preparation. For higher yield cases (> $10^6$), some cells were control frozen in liquid nitrogen as viable cells (and as a source of DNA) and an aliquot stored in GIT.

***Detection of TEL(ETV6)-AML1 (CBFA2)*** RNA was reverse transcribed to generate cDNA, the integrity of which was assessed by reverse transcriptase PCR (RT-PCR) amplification of a house-keeping gene (*c-ABL*). Screening for (*TEL-AML1*) by RT-PCR was with a single pair of primers as described previously (Romana et al, 1995). Fifty cases that had been investigated for evidence of the *TEL-AML1* fusion by RT-PCR (see below), were also independently tested by interphase fluorescence in situ hybridization (FISH) using dual colour probes (Vysis, UK) in the cytogenetics reference laboratory (C Harrison, Royal Free Hospital, London). FISH revealed six *TEL-AML*1-positive cases, which had not been detected by RT-PCR (and one case was negative by FISH, which had been found to be positive by RT-PCR).

RT-PCR was also used to screen for the t(1;19) (E2A-PBX), t(9;22) (*BCR-ABL*) translocations and for translocations of *MLL* with *AF4*, *AF6*, *AF9* and *ENL* (Repp et al, 1995). These tests used nested primers with high sensitivity ($10^{-4}/10^{-5}$). These latter screens produced an unacceptable level of false-positives (and some false-negatives) and therefore karyotype alone was used for the final molecular classification based upon these three types of translocations. Deletion of the *TAL* gene in cases of T-ALL was detected by direct PCR using a single set of primers as previously described (Janssen et al, 1993). A subset of ALL cases with high white cell counts were also assayed for deletion and methylation of the cell cycle inhibitor genes *CDKN2/p16*$^{INK4A}$ and *CDKN1/p15*$^{INK4B}$. These data were not, however, incorporated into the final molecular diagnostic scheme and are reported in detail elsewhere (Iravani et al, 1997).

***FISH detection of ploidy changes*** Cases that were registered as karyotypically normal or failed in the LRF/UK Cancer Cytogenetics Study Group Database (see above) were screened (by the molecular genetics reference laboratory) by interphase FISH for extra copies of chromosomes X and 21 using chromosome specific probes. The presence of extra copies of both X and 21 was required to define hyperdiploidy. These two chromosomes were selected as our pilot study and published reports (Moorman et al, 1996; Raimondi et al, 1996) indicated that > 90% of cases of ALL that are high hyperdiploid include additional copies of both 21 and X.

***Sample banking*** Material remaining on some patients after molecular screening included bone marrow smears, plasma and small quantities of cells, RNA, cDNA and DNA suitable for PCR-based assays. These have been stored for future studies.

### Results and discussion

A haematological and immunophenotypic subdivision of all patients eligible for the study and interviewed is given in Table 19. Non-Hodgkin's lymphoma data are given separately in Table 18 but we note here there is a biological and clinical case for T-NHL and T-ALL being subtle manifestations of essentially the same thymic or T-cell precursor malignancy. They may therefore be pooled together in later analyses. Similarly, rare cases of FABL3 Burkitt-like ALL (mature B immunophenotype) are more rationally grouped along with B-NHL.

Figures 7A, 7B and 8 show the breakdown of cases according to the major cytogenetic and molecular genetic abnormalities. Molecular genetic diversity in acute leukaemia is extensive; indeed in complete detail it is likely that every patient has a distinct molecular pattern of clonal diversity. A substantial number of patients had either infrequently occurring chromosomal abnormalities or highly complex karyotypes in which none of the common chromosomal abnormalities were identified; these were grouped together under 'Others'. Some chromosomal deletions appear to occur relatively commonly in different immunological subtypes and in conjunction with other molecular changes, for

 *© 2000 Cancer Research Campaign*

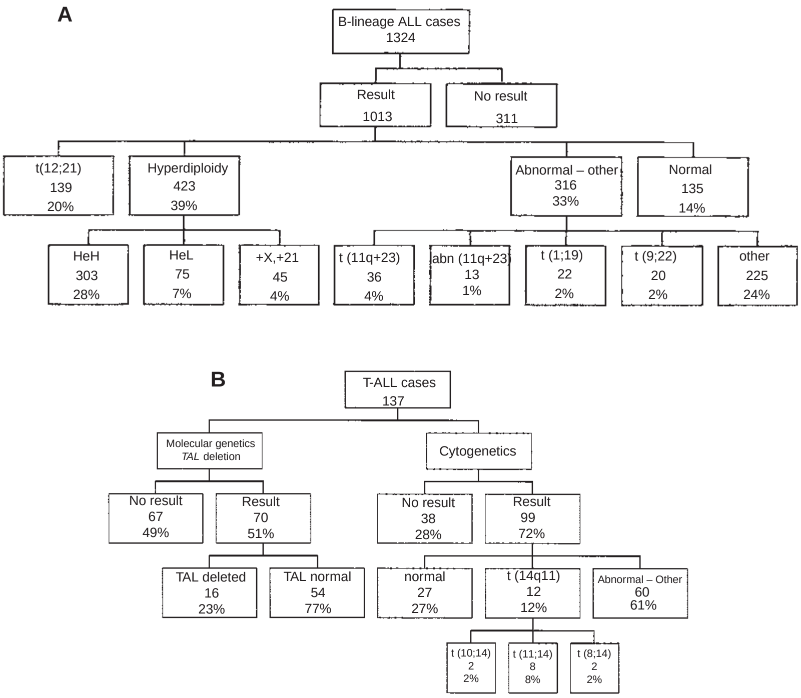

**Figure 7** Cytogenetic and molecular genetics classification of ALL cases: (**A**) B-lineage and lineage unknown; (**B**) T-lineage. Result, successful cytogenetic result or positive molecular result; Hyperdiploidy, 47 to 65 chromosomes; heH, 51–65 chromosomes; HeL, 47–50 chromosomes; '+X, +21', trisomies X and 21 by FISH (when cytogenetics was normal or failed). Denominators used to calculate percentages: 'Abnormal - other' and 'normal' branches: 949 - number of cases with a cytogenetic result; t(12:21):693 - number of cases tested for t(12:21) by PÇÂor FISH; Hyperdiploidy branch: 1073 – number of cases with a cytogenetics – result plus number tested for +X, +21 by FISH

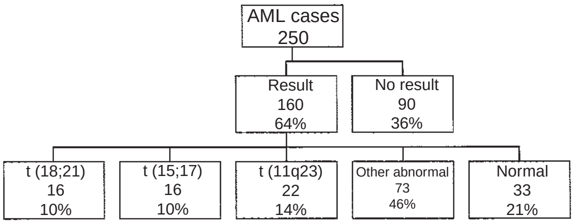

**Figure 8** Cytogenetic and molecular genetics classification of AML cases example deletion of the long arm of chromosome 6 [del(6q⁻)] or the short arms of chromosomes 9 and 12 [del(9p), del(12p)]. It is suspected, though unproven, that these may be secondary changes associated with disease progression and although we have these data available for subsequent analysis, we have not incorporated them here for classification purposes.

The numbers in some molecularly defined subgroups in this study are small which will restrain the statistical strength of any subsequent analyses; however, some highly selective associations may emerge. Two subgroups are of appreciable size – the c-ALL with either hyperdiploidy (423) or *TEL-AML1* fusion (139). These data should enable us to assess whether any epidemiological associations found for ALL are more pronounced within these molecularly defined subgroups.

### *Age and gender*

As age and gender have been shown to be of some prognostic importance in leukaemia clinical trials (Kersey, 1997) and some biologically defined subgroups are already known to have a marked age and/or gender bias (Greaves et al, 1985; Greaves, 1999), we also summarize here, in Figures 9–13, the age distributions within the UKCCS data set of the major biological subsets and in Tables 19 and 20 provide a breakdown according to gender and broad age groupings.

### MOLECULAR ANALYSIS OF GENETIC SUSCEPTIBILITY

There is now increasing recognition that genetic susceptibility can influence the risk of childhood cancer (Perera, 1997). Identification of genes that increase susceptibility might therefore help to pinpoint the role of specific carcinogens (Suk and Collman, 1998). To facilitate studies of genetic susceptibility, the UKCCS has involved the systematic collection of post-treatment blood samples from children with cancer, and, as healthy controls, from their parents, sibs and unrelated children. In the first instance the molecular analysis has involved comparisons of *HLA class II* (*DPB1, DQA1, DQB1*) allele frequencies in cases and controls for evidence of allele associations that could, in conjunction with epidemiological data, provide support for the role of infection in the aetiology of childhood leukaemia and lymphoma. Since no previous study of childhood cancer in the UK has involved the collection of post-treatment blood samples from children with cancer on a national scale, this paper presents details of the methods used, reviews sample collection, and provides brief details of the HLA typing methodology.

© 2000 Cancer Research Campaign 

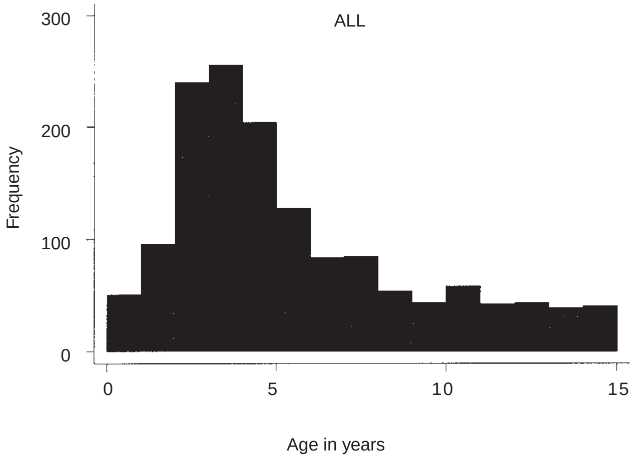

**Figure 9** Frequency distribution of all ALL cases by single years of age

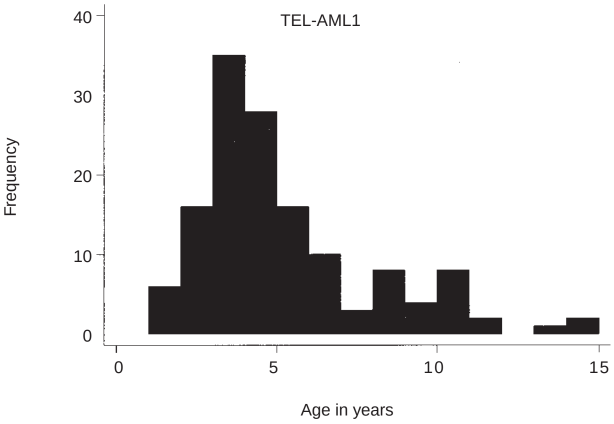

**Figure 10** Frequency distribution of TEL-AMLI cases by single years of age

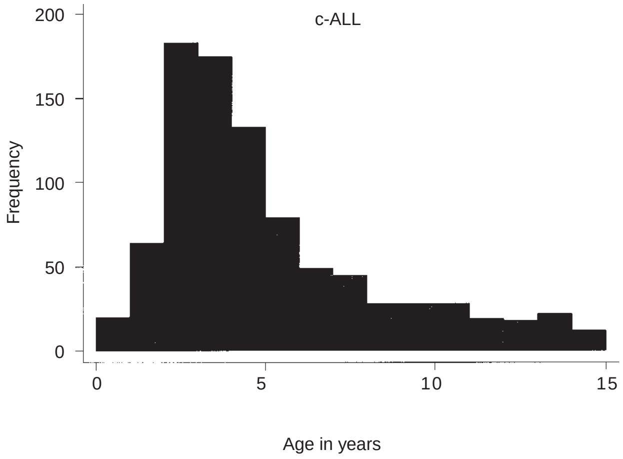

**Figure 11** Frequency distribution of C-ALL cases by single years of age

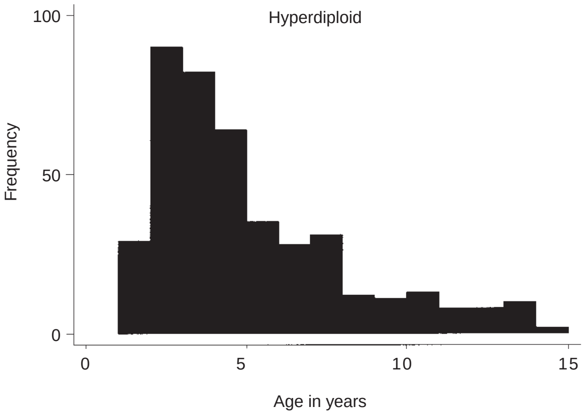

**Figure 12** Frequency distribution of hyperdiploidy cases by single years of age

**Table 20** Molecular cytogenetics in ALL cases (excluding those classified as T-ALL)

| | Hyperdiploid | *TEL-AML1* | Other abnormality | 'Normal' | Unknown | Overall total |
|---|---|---|---|---|---|---|
| Sex | | | | | | |
| Boys | 241 (57.0)[a] | 74 (53.2) | 157 (49.7) | 84 (62.2) | 174 (56.0) | 730 (55.1) |
| Girls | 182 (43.0) | 65 (46.8) | 159 (50.3) | 51 (37.8) | 137 (44.1) | 594 (44.9) |
| Age at diagnosis | | | | | | |
| 0 | 0 (0) | 0 (0) | 30 (9.5) | 2 (1.5) | 17 (5.5) | 49 (3.7) |
| 1 | 29 (6.9) | 6 (4.3) | 22 (7.0) | 9 (6.7) | 23 (7.4) | 89 (6.7) |
| 2–5 | 271 (64.1) | 95 (68.3) | 156 (49.4) | 84 (62.2) | 171 (55.0) | 777 (58.7) |
| 6–14 | 123 (29.1) | 38 (27.3) | 108 (34.2) | 40 (29.6) | 100 (32.2) | 409 (30.9) |
| Down's syndrome | 5 (1.2) | 1 (0.7) | 5 (1.6) | 6 (4.4) | 7 (2.3) | 24 (1.8) |
| Total | 423 | 139 | 316 | 135 | 311 | 1324 |

[a]Percentage of cases (in vertical column).

### Methods and results

### *Blood sample collection*

The methods used in the molecular epidemiological analysis are summarized in Figure 14. The systematic collection of post-treatment blood samples from children with cancer, their parents and siblings was carried out by UKCCS interviewers, in paediatric oncology clinics and by GPs. Factors affecting sample retrieval included patient survival, and parental consent. Samples were obtained in remission from children with leukaemia, and post-therapy from children with lymphoma and solid tumours, when white cell counts had recovered to provide sufficient DNA. Samples from children with leukaemia, lymphoma and cancer usually consisted of blood (~5 ml) in EDTA for the extraction of genomic DNA. Samples (~5 ml) from children with leukaemia and lymphoma were also collected in preservative-free heparin (~5 ml)

 *© 2000 Cancer Research Campaign*

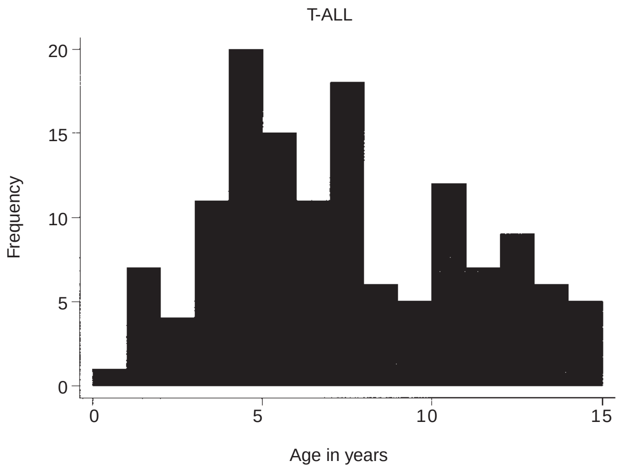

**Figure 13** Frequency distribution of T-ALL cases by single years of age

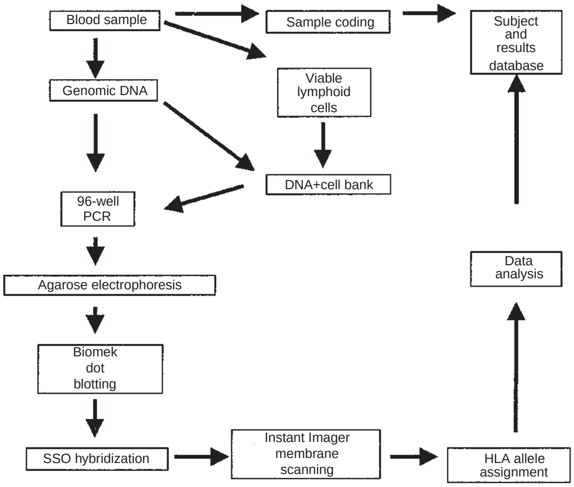

**Figure 14** Flow diagram showing steps in the molecular screening of index cases and controls for *HLA class II* alleles to retrieve viable lymphoid cells. Samples (~10 ml) from the parents and siblings of children with leukaemia or lymphoma were collected in the same way. Samples from the parents and siblings of children with solid tumours were collected in EDTA only.

Blood sample collection commenced in 1992, and continued until the end of 1997. The aim was to obtain as complete a set of samples from eligible index case children, and their parents and siblings as circumstances would allow. Genomic DNA was extracted from all blood samples since it provides the most robust and versatile source of biological material for the direct molecular analysis of genetic susceptibility. The PCR was used in the molecular analysis because it conserves DNA whilst allowing genetic screening to be carried out at high resolution. Since the amount of blood sample obtained was limited, viable lymphoid cells from children with leukaemia and lymphoma were frozen with the objective of preparing lymphoid cell-lines as an additional source of material.

### *Sample processing and storage*

Each child with cancer from whom a blood sample was received was assigned a unique laboratory code, and together with the child's name, diagnosis and other identifiers, this was entered into a Microsoft Access database. Information about relatives (parents, siblings) of the child with cancer from whom a sample was obtained was also recorded in the database. Genomic DNA was extracted from all blood samples using a resin-based commercial kit (BACC2, Nucleon), the DNA diluted to 50 ng $\mu l^{-1}$, and stored at – 80°C. Lymphoid cells were isolated from the blood of children with leukaemia and lymphoma and from their parents by centrifugation over Lymphoprep (Nycomed), and stored in a viable state in liquid nitrogen.

### *Blood samples from children with cancer*

Post-treatment blood samples were obtained from 1863 (48%) of the 3838 children eligible for the UKCCS. These included samples for 1183 (68%) of the 1736 children with leukaemia, 181 (51%) of the 349 children with lymphoma (Hodgkin's and non-Hodgkin's), and 499 (28%) of the 1753 children with solid tumours.

### *Family controls*

Where possible, blood samples were collected from both biological parents of each child with cancer for the purpose of providing family-based controls. Samples were obtained from both parents of 846 (48%) of the eligible children with leukaemia, 131 (37%) of those with lymphoma and 301 (17%) of those with solid tumours. Blood samples were obtained from the healthy sibs of 719 of the children with cancer. These included 426 sibs of children with leukaemia, 91 sibs of children with lymphoma and 202 sibs of children with solid tumours.

### *Unrelated childhood controls*

Ethical considerations precluded the collection of blood samples from UKCCS case-matched control children. Children with other types of cancer were therefore used as controls for any specified type of cancer (for example, children with solid tumours served as controls for children with leukaemia). In the absence of an adequate national sample of case-matched control children without cancer, data from 1500 full-term healthy newborn babies delivered in St Mary's Hospital, Manchester were additionally used as a provisional comparison group.

### *Molecular analysis*

The molecular analysis (Figure 14) set out to compare allele, phenotype, and haplotype frequencies between children with different types of cancer, and with parental, sib or unrelated controls at three *HLA class II* loci (*DPB1, DQA1, DQB1*) loci. Using genomic DNA, exon 2 of *HLA-DPB1, DQA1 or DQB1* was amplified in the polymerase chain reaction using locus-specific primers (Fernandez-Vina and Bignon, 1997). The amplified PCR products were arrayed at high density by dot-blotting onto replicate nylon membranes using a Beckman Biomek 2000, and the membranes hybridized with $^{32}$P-labelled sequence specific oligonucleotides (SSO). The membranes were scanned for hybridization with SSO probes on a Packard InstantImager, and alleles assigned to each individual based on patterns of positive and negative SSO probe reactions for each locus.

The *HLA-DPB1*, *DQA1* and *DQB1* type of each subject was stored in the Microsoft Access database, and data relevant for the analysis of *HLA* associations collated in Access Queries for output into Microsoft Excel files. These were used to compute allele, genotype and phenotype frequencies for each diagnostic

© 2000 Cancer Research Campaign case and control series. Associations with specific *HLA* alleles were expressed as odds ratios with 95% confidence intervals (CI), and the significance of differences between different groups in Fisher's two-sided exact test, with appropriate Bonferroni corrections.

### Discussion

It is increasingly recognized that the aetiology of childhood cancer involves pre- or early post-natal exposure to environmental carcinogens and a host response modulated both by developmental and inter-individual genetic variations. The identification of constitutional variations in specific genes involved in the metabolism of carcinogens offers a way of predicting the aetiological role of a given environmental factor.

The UKCCS has included the systematic collection of post-treatment blood samples from children with cancer and from related and unrelated controls for molecular studies of variations in cancer susceptibility. The primary aim of these studies was to analyse the frequency of *HLA class II* alleles in childhood leukaemia and lymphoma. An allele association could be construed as supporting the hypothesis of an immune-mediated, possibly infectious, aetiology in childhood leukaemia/lymphoma (Greaves, 1999). Existing evidence for the role of *HLA class II* alleles in susceptibility to childhood leukaemia and lymphoma is limited to small patient series (Taylor et al, 1998). Although the UKCCS has involved much larger patient and control series, logistic considerations required the development of a high-throughput HLA typing method. This included the batchwise amplification with *HLA* locus-specific PCR primers and the analysis of SSO hybridization by automated scanning.

The molecular screening of genetic variations in cases compared with controls is becoming a more widely accepted part of epidemiological studies. Molecular methods are able to identify inter-individual genetic variations that may influence childhood cancer susceptibility with remarkable precision. The intrinsically difficult assessment of environmental exposures in relation to the risk of childhood cancer is thus complemented by data on inter-individual genetic variations in carcinogen-response genes that may help to define susceptible patient groups. Careful selection of candidate genes with known functions and sound aetiological hypotheses reduces the empirical nature of this analysis. The development of high-throughput micro array-based assays offers an opportunity for testing the role of given genetic variations in statistically robust patient and control groups. The collection of a comprehensive series of post-treatment blood samples from children with cancer as part of the UKCCS shows that this can be achieved as part of a comprehensive epidemiological study.

### UK CHILDHOOD CANCER STUDY INVESTIGATORS

### UKCCS Region

**Central** *Department of Public Health & Epidemiology, University of Birmingham:* A Boulton, P Boyd, KK Cheng[1], J Cook, EA Gilman[2], D Lunt, H Mahler†, C Walker, M Wardroper; *Birmingham Children's Hospital:* PJ Darbyshire, FGH Hill, JR Mann, B Morland, F Raafat, MCG Stevens

**E Anglia** *Department of Community Medicine, University of Cambridge:* A Ahmed, P Amos, V Bone, S Bonney, M Bray, P Cambouropoulos, S Cook, N Day[1], S Elkins, F Hensel, P Lucas, J Pettinger, M Pugsley, E Ruja, J Skinner, D Williams; *Addenbrooke's Hospital, Cambridge:* V Broadbent, M Williams

**N East** *Leukaemia Research Fund Centre for Clinical Epidemiology, Leeds:* M Alcock, K Bell, M Buchan, R Cartwright[1], H Cusack, N Fear, S Griffiths, J Jarvis, P Johnson, E Kane, G Law, A Moorman, J Prajapati, P Roberts, E Roman[1], J Simpson, V Sinclair, A Staines, C Thackrah, S Thistlethwaite, B Waller; *St James's University Hospital, Leeds:* C Bailey, S Kinsey, I Lewis, S Picton, R Squire, R Taylor; *Leeds General Infirmary:* JM Beck, RML Doran, JH Livingston, P Van Hille; *Hull Royal Infirmary*: I Beddis; *Pinderfields General Infirmary*: MM Cameron; *Royal Victoria Infirmary, Newcastle*: A Craft[1], J Hale, J Kernahan, M Reid, K Windebank; *Department of Child Health, Newcastle*: A Pearson, R Skinner; *Middlesbrough General Hospital*: S Marks

**N West** *Royal Manchester Children's Hospital, University of Manchester*: J Achilles, S Alam, JM Birch[1], V Blair, B Buckley, M Clarkson, OB Eden[1], S Howell, C Kellaway, L Lashford, S Leeke, P Leggett, AV Murphy, C O'Rorke, S Panton, J Paxon, H Pots, C Roberts, J Rothwell, W Stephenson, B Whelpton; *Alder Hey Children's Hospital*: M Caswell, H McDowell, BL Pizer; *Christie Hospital, Manchester*: R Gattamaneri; *Clatterbridge Hospital, Liverpool*: *J* Brock; *Royal Manchester Children's Hospital*: AM Kelsey, R Stevens, A Will, B Brennan

**Scotland** *Information & Statistics Division, Common Services Agency for the National Health Service in Scotland:* J Brydon, C Dodds, E Findlay, J Finucane, J Fraser, E Harkness, A Heary, N Hunter, E Juszczak, M Lang, E Lapsley, A McArthur, A MacCalman, PA McKinney[1], K Proudfoot, C Smith, K Smith, D Stockton, CS Thomson, R Vickers; *Ninewells Hospital, Dundee:* R Wilkie; *Royal Aberdeen Children's Hospital, Aberdeen:* D King; *Royal Hospital for Sick Children, Edinburgh:* G Mackinlay, P Shaw, A Thomas, H Wallace; *Royal Hospital for Sick Children, Yorkhill, Glasgow;* R Carachi, BS Gibson, E Simpson; *Southern General Hospital, Glasgow;* G Cruickshank, TAH Hide; *Western General Hospital, Edinburgh:* A Gregor, AJW Steers; *Western Infirmary, Glasgow:* A Barrett, DL Hamblen, SB Kaye, R Mackie

**S East** *Institute of Cancer Research, Section of Epidemiology:* A Allen, A Arden Jones, S Beeby, V Bignall, L Breeze, J Deacon[2], M MacDonald, F Matthews, C Meggitt, J Peto[1], E Sharpe, C Spencer, J Swales, M Thorne, P Trowbridge, J Webster-King, E Williams; *Atkinson Morley's Hospital:* BA Bell, FG Johnston, HT Marsh, D Uttley; *Brook Hospital:* J Bartlett, A Evans, RW Gullan; *Charing Cross Hospital:* MG Glaser, D Peterson, BM Southcott; *Chelsea and Westminster Hospital:* N Cavanagh; *Conquest Hospital:* K Pearl, D Scott; *Farnborough Hospital:* CW Darby; *Great Ormond Street Hospital for Children:* J Chessels, J Evans, M Gaze, IM

 © 2000 Cancer Research Campaign

Hann, W Harkness, R Hayward, A Michalski, J Passmore, M Phillips, J Pritchard; *Guy's Hospital:* KGA Clark, EA MacDonald, BGR Neville, SA Robb, RO Robinson; *Hurstwood Park Neurological Centre:* C Hardwidge; *Kent and Canterbury Hospital:* N Padgham; *Kent County Ophthalmic and Aural Hospital:* VJ Lobo; *Maidstone Hospital:* C Keen; *Middlesex Hospital:* PC Hindmarsh, AM Kilby, RL Souhami; *Moorfields Eye Hospital:* S Tuft; *Northwick Park Hospital:* RM Thomas; *Princess Royal Hospital:* P Ward; *Queen Mary's University Hospital, Roehampton:* M Scott; *Royal Free Hospital:* AV Hoffbrand, HG Prentice; *Royal London Hospital:* CG Gutteridge, AC Newland; *Royal Marsden Hospital:* M Brada, JM Henk, S Meller, R Pinkerton, K Pritchard Jones; *Royal National Orthopaedic Hospital:* S Cannon; *Royal Sussex County Hospital:* DS Murrell; *St Bartholomew's Hospital:* JL Hungerford, JE Kingston, PN Plowman, B Young[2]; *St George's Hospital:* SE Ball, SNJ Capps, EG Davies, SJK Holmes; *St Thomas's Hospital:* R Carr, DM Mercer, MA Smith; *West Hill Hospital:* VE Andrews; *West Middlesex University Hospital:* RG Hughes

**S Midlands** *Imperial Cancer Research Fund Cancer Epidemiology Unit, University of Oxford:* P Ansell, K Baker, V Beral[1], J Black, S Boon, C Burge, F Burge, A Cliff, D Deciaccio, P Dorman, F Heydon, N Langley, M Pelerin, J Roemmele, K Sayers, P Townshend; *Central Middlesex Hospital:* S Harman, J Loftus; *Edgeware General Hospital*: S Roth; *Hemel Hempstead Hospital*: B Lee; *Hillingdon Hospital:* R Buchdahl; *John Radcliffe Hospital, Oxford*: DB Dunger, C Mitchell, MKM Moncrieff, PKH Tam, K Wheeler; *Lister Hospital, Stevenage*: J Reiser; *Milton Keynes General Hospital:* V Joss, DJ Moir; *North Hampshire Hospital, Basingstoke*: J Darmady; *Northampton General Hospital*: P Daish; *Northwick Park Hospital*: MM Liberman†; *Queen Elizabeth II Hospital, Welwyn Garden City*: MS Al-Izzi; *Radcliffe Infirmary, Oxford*: CBT Adams, RSC Kerr, PJ Teddy; *Royal Berkshire Hospital, Reading*: CJ Barton, CL Newman; *St Albans City Hospital*: CM Gabriel; *Stoke Mandeville Hospital, Aylesbury*: M O'Hea, S Sherrin, A Watson; *Watford General Hospital*: E Douek; *Wexham Park Hospital, Slough*: JA Connell; *Wycombe General Hospital*: CH Cheetham, S Kelly

**S Wales** *Medical Research Council's Epidemiology Unit*: A Beswick, B Eldridge, P Elwood[1], J Hughes; *South Glamorgan Health Authority*: M Phillips, D Webb

**S West** *University of Edinburgh:* FE Alexander[1], *University of Southampton, Wessex Medical Oncology Unit:* B Bennett-Lloyd, A Davis, R Dunn, J Little, S Longdon, M Mitchell, S Muir, J Sturitis; *Southampton University Hospitals NHS Trust:* C Kennedy, J Kohler, D Lang, M Radford; *Bristol Royal Hospital for Sick Children:* N Foreman, A Foot, M Mott[2], H Noblett, A Oakhill; *Frenchay Healthcare NHS Trust, Bristol:* D Sandeman; *Freedom Fields Hospital, Plymouth:* J Baumer, P Ward; *Royal Devon and Exeter Healthcare NHS Trust:*, A McNinch; *Royal Cornwall Hospitals NHS Trust, Truro:* N Gilbertson; *North Devon and District Hospital, Barnstaple:* A Bosley, S Richardson; *Taunton & Somerset Hospital, Musgrave Park:* D Challacombe, T French

**Trent** *University of Nottingham Medical School:* L Bate, CED Chilvers[1], G Faulkner, P Hawtin, C Jenkinson, P Kelham, I Mackie, M Mackie, KR Muir[2], J O'Dwyer, A Williams; *Derby Children's Hospital:* C Nelson; *Leicester Royal Infirmary:* C Howarth, M Madi, R Shannon; *Queen's Medical Centre Nottingham:* K Forman, M Hewitt, J Punt, D Walker; *Sheffield Children's Hospital:* M Gerrard, JS Lilleyman, A Vora

*Childhood Cancer Research Group, University of Oxford*: G Draper

*Cytogenetics Laboratory, Haematology Department, Royal Free Hospital*: C Harrison

*Epidemiological Studies Unit, University of Oxford*: R Doll[1], S Richards

*Immunogenetics Laboratory, University of Manchester*: M Ayres, R Carter, SP Dearden, A Hussain, J Kennedy, P Ravetto, A Ruprai, GM Taylor[1], J Taylor, PD Watson

*Leukaemia Research Fund Centre, Institute of Cancer Research*: SM Colman, MF Greaves[1], CM Price

*Medical Research Council Radiation and Genome Stability Unit*: DT Goodhead[1]

*National Radiological Protection Board*: S Allen, D Bartlett, RP Blackwell, F Fry[1], M Maslanyj, T Mee, J Miles

*UK Coordinating Committee for Cancer Research*: G Adams[1,†]

Writing Committees: **Introduction** CED Chilvers[1], R Doll[1]; **Material & General Methodology** G Law, E Roman[1], J Simpson; **Electromagnetic Fields** S Allen, N Day[1], M Maslanyj, T Mee, E Roman[1], J Skinner; **Radon and Terrestrial Gamma Radiation** R Cartwright[1], G Law, E Roman[1]; **Diagnostic Review and Classification of Solid Tumours** JM Birch[1], AM Kelsey; **Leukaemia: Biological Samples and Classification** OB Eden[1], MF Greaves[1], CJ Harrison, A Moorman, E Roman[1], J Simpson; **Molecular Analysis of Genetic Susceptibility** FE Alexander[1], JM Birch[1], OB Eden[1], J Simpson, GM Taylor[1]

[1]Management Committee. [2]Co-opted member of biological or epidemiological subcommittee of Management Committee. †Deceased

### ACKNOWLEDGEMENTS

The success of the UKCCS has depended on the help of many thousands of people, most notably the parents of nearly 4000 children, who have had the tragic experience of seeing their children develop cancer, and those of nearly 8000 healthy children, all of whom have given their time to answer long questionnaires; we are most grateful for their cooperation. We are grateful, too, for the assistance provided by thousands of general practitioners who have allowed us to examine their records; members of the UK Childhood Cancer Study group and other clinicians whose names have not been listed above, but who have notified us of children under their care and allowed us to interview their patients and obtain, in many cases, samples of blood and bone marrow; Mary Martineau and the members of the UK Cancer Cytogenetics Group, Marjan Iravani, Ruby Dhat and Abiromi Anonda;

David Gokhali, Mark Robinson and Carolyn Watson who assisted in the study of immunogenetics; the staff of the Regional Cancer Registries who helped ensure case ascertainment and of the FHSAs and HBs who helped with the selection of control children; the members of the UKCCCR staff, most notably Peter Twentyman, Jean Mossman, Stephanie Ashby and Joanne Bull who have in different capacities controlled expenditure on the study and serviced the work of the Management Committee; Barbara Deverson and Cathy Harwood, who prepared manuscripts of this report, and the many clerical and secretarial staff, not listed above, who have helped in recording the study data at different times over 8 years. We acknowledge above all our debt to Professor Ged Adams, one time Chairman of the Radiation Subcommittee of the UKCCCR whose inspiring and gentle leadership brought and kept together such a diverse group of investigators, but who died shortly before any results of the study were known.

### FUNDING

Funding for the study has been provided by the Cancer Research Campaign, the Imperial Cancer Research Fund, the Leukaemia Research Fund, and the Medical Research Council through grants to their units and through the UKCCCR, by grants from the Department of Health, the Electricity Association, the Leukaemia Research Fund, the Irish Electricity Supply Board, the National Grid and Westlakes Research (Trading) for the general expenses of the study; by the Kay Kendall Leukaemia Fund and the Medical Research Council for the associated laboratory studies and by the Foundation for Children with Leukaemia for the study of electrical fields. The investigation in Scotland has been principally funded separately by the Scottish Office Home & Health Department and by Scottish Power plc, Scottish Hydro-Electric plc and Scottish Nuclear plc.

### REFERENCES

Advisory Group on Non-ionising Radiation (1992) Electromagnetic fields and the risk of cancer. *Doc NRPB*, **3**(1), pp. 1–138. National Radiological Protection Board: Chilton

Advisory Group on Non-ionising Radiation (1994) Electromagnetic fields and the risk of cancer. *Doc NRPB*, **5**(2), pp. 79–81. National Radiological Protection Board: Chilton

Arundel SE and Kinnier-Wilson LM (1986) Parental occupations and cancer: a review of the literature. *J Epidemiol Comm Hlth* **40**: 30–36

BEIR VI (1999) *Health Risks of exposure to Radon.* National Academy Press: Washington

Berg JW (1996) Morphologic classification of human cancer. In: *Cancer Epidemiology and Prevention*, Schottenfeld D and Fraumeni JF (eds), pp. 28–44. Oxford University Press: New York

Bernard OA, Romana SP, Poirel H and Berger R (1996) Molecular cytogenetics of t(12;21)(p 13;q22). *Leuk Lymphoma* **23**: 459–465

Birch JM and Marsden HB (1987) A classification scheme for childhood cancer. *Int J Cancer* **40**: 620–624

Botting B and Crawley R (1995) Trends and patterns in childhood mortality and morbidity. In: *The Health of our Children. Decennial supplement*, Botting B (ed), pp. 62–81. OPCS Series DS No. 11

Bunin GR, Kramer S, Marrero O and Meadows AT (1987) Gestational risk factors for Wilms' tumor: results of a case–control study. *Cancer Res*. **47**: 2972–2977

Catovsky D, Matutes E, Buccheri V, Shetty V, Hanslip J, Yoshida N and Morilla R (1991) A classification of acute leukaemia for the 1990s. *Ann Hemat* **62**: 16–21

Census (1991) *The 1991 Census*. Crown copyright. ESRC purchase.

Central Statistical Office (1992) *Standard Industrial Classification of Economic Activities*. HMSO: London

Coleman MP, Babb P, Dameicki P, Grosclaude P, Honjo S, Jones J, Knerer G, Pitard A, Quinn M, Sloggett A and De Stavola B (1999) *Cancer Survival Trends in England & Wales, 1971–1995: Deprivation and NHS Region. Studies in Medical and Population Subjects 61*. Office of National Statistics: London

Darby S, Whitley E, Silcocks P, Thakrar B, Green M, Lomas P, Miles J, Reeves G, Fearn T and Doll R (1998) Risk of lung cancer associated with residential radon exposure in southwest England: a case–control study. *Br J Cancer* **78**(3): 394–408

Dockerty JD, Elwood JM, Skegg DCG and Herbison GPH (1998) Electromagnetic field exposures and childhood cancers in New Zealand. *Cancer Causes Control*, **9**: 299–309

Draper G (1991) *The Geographical Epidemiology of Childhood Leukaemia and Non-Hodgkin's Lymphoma in Great Britain 1966–83*. OPCS: London

Draper G (1995) Cancer. In: *The Health of our Children. Decennial supplement*, Botting B (ed), pp. 135–147. OPCS series DS No. 11

Fear NT, Roman E, Reeves G and Pannett B (1999) Father's occupation and childhood mortality: analysis of routinely collected data. *Health Stat Quart* **2**: 7–15

Fernandez-Vina MA and Bignon JD (1997) Primers and oligonucleotide probes (SSOP) used for DNA typing of HLA class II alleles. In: *Genetic Diversity of HLA. Functional and Medical Implication*, Charron D (ed), volume 1. EDK: Paris

FoxPro Users Guide (1991) Perrysburg, Ohio: Fox holdings

Gardner MJ, Snee MP, Hall AJ, Powell CA, Downes S and Terrell JD (1990) Results of case-control study of leukaemia and lymphoma among young people near Sellafield nuclear plant in West Cumbria. *Br Med J* **300**: 423–429

Gold EB and Gordis L (1978) Patterns of incidence of brain tumors in children. *Ann Neurol* **5**: 565–568

Gold E, Gordis L, Tonascia J and Szklo M (1979) Risk factors for brain tumors in children. *Am J Epidemiol* **109**: 309–319

Golding J, Paterson M and Kinlen LJ (1990) Factors associated with child cancer in a national cohort study. *Br J Cancer* **62**: 304–308

Greaves M (1988) Speculations on the cause of childhood acute lymphoblastic leukaemia. *Leukaemia* **2**: 120–125

Greaves M (1996*a*) The new biology of leukemia. In: *Leukemia*, Henderson ES, Lister TA and Greaves MF (eds), pp. 34–45. WB Saunders: Philadelphia

Greaves MF (1996*b*) Workshop Report. Infant leukaemia biology, aetiology and treatment. *Leukemia* **10**: 372–377

Greaves MF (1997) Aetiology of acute leukaemia. *Lancet* **349**: 344–349

Greaves MF (1999) Molecular genetics, natural history and the demise of childhood leukaemia. *Eur J Cancer* **35**: 173–185

Greaves MF, Pegram SM and Chan LC (1985) Collaborative group study of the epidemiology of acute lymphoblastic leukaemia subtypes: background and first report. *Leuk Res* **9**: 715–733

Greaves MF, Colman SM, Beard MEJ, Bradstock K, Cabrera ME, Chen P-M, Jacobs P, Lam-Po-Tang PRL, MacDougall LG, Williams CKO and Alexander FE (1993) Geographical distribution of acute lymphoblastic leukaemia subtypes: second report of the collaborative group study. *Leukemia* **7**: 27–34

Grufferman S, Wang HH, Delong ER, Kimm SYS, Delzell ES and Falletta JM (1982) Environmental factors in the etiology of rhabdomyosarcoma in childhood. *J Natl Cancer Inst* **68**: 107

Gunby JA, Darby SC, Miles JCH, Green BMR and Cox DR (1993) Factors affecting indoor radon concentration in the United Kingdom. *Health Phys* **64**: 2–12

Gutensohn NM and Cole P (1981) Childhood social environment and Hodgkin's disease. *N Engl J Med* **304**: 135–140

Gutensohn NM and Shapiro DS (1982) Social class risk factors among children with Hodgkin's disease. *Int J Cancer* **30**: 433–435

Hann I, Richards SM, Eden OB and Hill FGH (1998) Analysis of the immunophenotype of children treated on the Medical Research Council UK ALL Trial XI (MRC UKALLXI). *Leukemia* **12**: 1249–1255

Hartley AL, Birch JM, McKinney PA, Teare MD, Blair V, Carette J, Mann JR, Draper GJ, Stiller CA, Johnston HE, Cartwright RA and Waterhouse JAH (1998) The inter-regional epidemiology study of childhood cancer (IRESCC): case–control study of children with bone and soft tissue sarcomas. *Br J Cancer* **58**: 838–842

Henderson ES, Lister TA and Greaves MF (eds) (1996) *Leukemia*, p. 619. WB Saunders: Philadelphia

Henshaw DL, Eatough JP and Richardson RB (1990) Radon as a causative factor in induction of myeloid leukaemia and other cancers in adults and children. *Lancet* **335**: 1008–1012

Hughes JS and O'Riordan MC (1998) *Radiation Exposure of the UK Population –1993 review*. NRPB-R263. NRPB: Oxon

Iravani M, Dhat R and Price CM (1997) Methylation of the multi tumor suppressor gene-2 (MTS2, CDKN1, $p15^{ink4b}$) in childhood acute lymphoblastic leukemia. *Oncogene* **15**: 2609–2614

 *© 2000 Cancer Research Campaign*

ISCN (1995) *An International System for Human Cytogenetic Nomenclature*. S Karger: Basel
Janssen JWG, Ludwig W-D, Sterry W and Bartram CR (1993) SIL-TAL 1 deletion in T-cell acute lymphoblastic leukemia. *Leukemia* **7**: 1204–1210
Kaune WT, Feychting M, Ahlbolm A, Ulrich RM and Savitz DA (1998) Temporal characteristics of transmission-line loading in the Swedish Childhood Cancer Study. *Bioelectromagnetics* **19**: 354–365
Kendall GM, Miles JCH, Cliff KD, Green BMR, Muirhead CR, Dixon DW, Lomas PR and Goodridge PM (1998) *Exposure to Radon in UK Dwellings*. NRPB-R272. National Radiological Protection Board, Chilton
Kersey JH (1997) Fifty years of studies of the biology and therapy of childhood leukemia. *Blood* **90**: 4243–4251
Kinlen L (1988) Evidence for an infective cause of childhood leukaemia: comparison of a Scottish new town with nuclear reprocessing sites in Britain. *Lancet* **2**: 1323–1326
Kinlen L, Clarke K and Hudson C (1990) Evidence from population mixing in British new towns 1946–85 of an infective basis for childhood leukaemia. *Lancet* **336**: 577–582
Kleinerman RA, Linet MS, Hatch EF, Wacholder S, Tarone RE, Severson RK, Kaune WT, Friedman DR, Haines CM, Muirhead CR, Boice JD and Robison LL (1997) Magnetic field exposure assessment in a case–control study of childhood leukaemia. *Epidemiology* **8**: 575–583
Kneale GW, Stewart AM and Kinnear Wilson AM (1986) Immunization against infectious diseases in childhood cancer. *Cancer Immunol Immunother* **21**: 129–132
Knox EG, Stewart AM and Kneale GW (1983) Foetal infection, childhood leukaemia and cancer. *Br J Cancer* **48**: 849–852
Kramer S, Ward E, Meadows AT and Malone KE (1987) Medical and drug risk factors associated with neuroblastoma: a case–control study. *J Natl Cancer Inst* **78**: 797–804
Lilleyman JS, Hann IM, Stevens RF, Richards SM, Eden OB, Chessells JM and Bailey CC (1992) Cytomorphology of childhood lymphoblastic leukaemia: a prospective study of 2000 patients. *Br J Haematol* **81**: 52–57
Linet MS, Hatch EE, Kleinerman RA, Robison LL, Kaune WT, Friedman DR, Severson RK, Haines CM, Hartstock CT, Niwa S, Wacholder S and Tarone RE (1997) Residential exposure to magnetic fields and acute lymphoblastic leukemia in children. *N Engl J Med* **337**: 1–7
Lipson A and Bale P (1985) Ependymoblastoma associated with prenatal exposure to diphenylhydantoin and methylphenobarbitone. *Cancer* **55**: 1859–1862
Little J (1999) *The Epidemiology of Childhood Cancer*. IARC Scientific Publications No. 149. International Agency for Research on Cancer: Lyon
Lowengart RA, Peters JM, Cicioni C, Buckley J, Bernstein L, Preston-Martin S, Rappaport E (1987) Childhood leukaemia and parents' occupational and home exposures. *J Natl Cancer Inst* **79**: 39–46
McBride ML, Gallagher RP, Thériault G, Armstrong BG, Tamaro S, Spinelli JJ, Deadman JE, Fincham S, Robson D and Choi W (1999) Power-frequency electric and magnetic fields and risk of childhood leukaemia in Canada. *Am J Epidemiol* **149**: 831–842
McKinney PA, Cartwright RA, Saiu JMT, Mann JR, Stiller CA, Draper GJ, Hartley AL, Hopton PA, Birch JM, Waterhouse JA and Johnston HE (1987) A case–control study of aetiological factors in childhood leukaemia and lymphoma. *Arch Dis Child* **62**: 279–287
McKinney PA, Smith K and Findlay E (1995) The Scottish case–control study of childhood leukaemia and cancer: methodology and environmental measures of exposure. *Health Bull* **53**: 222–228
McKinney PA, Juszczak E, Findlay E and Smith K (1999) Pre- and perinatal risk factors for childhood leukaemia and other malignancies: a Scottish case–control study. *Br J Cancer* **80**: 1844–1851
Magee PN, Montesano R and Preussmann R (1976) N-nitroso compounds and related carcinogens. In: *Chemical Carcinogens*, Searle CE (ed), pp. 491–625. American Chemical Society: Washington DC
Merchant CJ, Renew DC and Swanson J (1994*a*) Origins and magnitudes of exposure to power frequency magnetic fields in the UK. *CIGRÉ*, 36–105
Merchant CJ, Renew DC and Swanson J (1994*b*) Exposures to power-frequency magnetic fields in the home. *J Radiol Prot* **14**: 77–87
Michaelis J, Schüz J, Meinert R, Zemann E, Grigat J-P, Kaatsch P, Kaletsch U, Miesner A, Brinkmann K, Kalkner W and Kärner H (1977) Combined risk estimates for two German population-based case–control studies on residential magnetic fields and childhood acute leukemia. *Epidemiology* **9**: 92–94
Miles JCH, Green BMR and Lomas PR (1996) Radon affected areas: England. *Doc. NRPB* **7**: 3–18. National Radiological Protection Board, Chilton
Miller RW, Young JL and Novakovic B (1995) Childhood cancer. *Cancer* **75** (1 Suppl): 395–405
Moorman A, Clark R, Farrell D, Hawkins J, Martineau J and Secker-Walker L (1996) Probes for hidden hyperploidy in acute lymphoblastic leukaemia. *Genes Chromosomes Cancer* **16**: 40–45
Mott MG, Mann JR and Stiller CA (1997) The United Kingdom Children's Cancer Study Group – the first 20 years of growth and development. *Eur J Cancer* **33**: 1448–1452
MRC (1997) *Annual Leukaemia Report. Childhood Trials*. MRC: London
MRC (1998) *Annual Leukaemia Report. Childhood Trials*. MRC: London
Office of National Statistics (1998) Simplified list of Social Class based on occupation: excluding employment status axis. OOSS User Guide: 6.1
Office of Population Censuses and Surveys (1990) *Standard Occupational Classification*, Vols 1–3. HMSO: London
Page H (1991) The use of the FHSA registration index for a health needs survey. *Hlth Services Management Res* **4**: 140–147
Parkin DM, Kramarova E, Draper GJ, Masuyer E, Michaelis J, Neglia J, Qureshi S and Stiller CA (1998) *International Incidence of Childhood Cancer*, vol. II. IARC Scientific Publications No. 144. International Association of Research on Cancer: Lyon
Perera FP (1997) Environment and cancer: who are susceptible? *Science* **278**: 1068–1073
Pinel J, Fearn T, Darby SC and Miles JCH (1995) Seasonal correction factors for indoor radon measurements in the United Kingdom. *Rad Prot Dosimetry* **58**: 127–132
Preece AW, Grainger O, Golding J and Kaune W (1996) Domestic magnetic field exposure in Avon. *Phys Med Biol* **41**: 71–81
Preston-Martin S, Yu MC, Benton B and Henderson BE (1982) N-nitroso compounds and childhood brain tumors: a case-control study. *Cancer Res* **42**: 5240–5245
Pui C-H and Evans WE (1998) Acute lymphoblastic leukemia. *N Engl J Med* **33**9: 605–615
Pui C-H, Behm GF and Crist WM (1993) Clinical and biologic relevance of immunologic marker studies in childhood acute lymphoblastic leukemia. *Blood* 82: 343–362
Pui C-H, Kane JR and Crist WM (1995) Biology and treatment of infant leukemias. *Leukemia* **9**: 762–769
QuickAddress (1999) Version 3.0 QAS Systems, London
Raimondi SC (1993) Current status of cytogenetic research in childhood acute lymphoblastic leukemia. *Blood* **81**: 2237–2251
Raimondi SC, Pui G-H, Hancock ML, Behm FG, Filatov L and Rivera GK (1996) Heterogeneity of hyperdiploid (51–67) childhood acute lymphoblastic leukemia. *Leukemia* **10**: 213–224
RCGP (1987) *Profile of UK Practices*. RCGP Information Sheet No. 2. Royal College of Practitioners: London
Repp R, Borkhardt A, Haupt E, Kreuder J, Brettreich S, Hammermann J, Nishida K, Harbott J and Lampert F (1995) Detection of four different 11q23 chromosomal abnormalities by multiplex-PCR and fluorescence-based automatic DNA-fragment analysis. *Leukemia* **9**: 210–215
Roberts HR, Rushton L, Muir KR, Dengler R, Coupland CAC, Jenkinson CM, Ruffell A and Chilvers CED (1995) The use of family health service authority registers as a sampling frame in the UK: a review of theory and practice. *J Epidemiol Comm Hlth* **49**: 344–347
Robison LL, Buckley JD, Daigle AE, Wells R, Benjamin D, Arthur DC and Denman-Hammond G (1989) Maternal drug use and risk of childhood nonlymphoblastic leukemia among offspring. *Cancer* **63**: 1904–1911
Roman E, Ansell P and Bull D (1997) Leukaemia and non-Hodgkin's lymphoma in children and young adults: are prenatal and neonatal factors important determinants of disease? *Br J Cancer* **74**: 406–415
Romana SP, Poirel H, Leconiat M, Flexor M-A, Mauchauffe M, Jonveaux P, Macintyre EA, Berger R and Bernard OA (1995) High frequency of t(12;21) in childhood B-lineage acute lymphoblastic leukemia. *Blood* **86**: 4263–4269
Ross JA, Davies SM, Potter JD and Robison LL (1994*a*) Epidemiology of childhood leukemia, with a focus on infants. *Epidemiol Rev* **16**: 243–272
Ross JA, Potter JD and Robison LL (1994*b*) Infant leukemia, topoisomerase II inhibitors, and the MLL gene. *J Natl Cancer Inst* **86**: 1678–1680
Secker-Walker LM (1997) *Chromosomes and Genes in Acute Lymphoblastic Leukaemia*. Chapman & Hall: New York
Shu X-O, Gao Y-T, Linet MS, Brinton LA, Gao BA, Jin F and Fraumeni JF Jr (1987) Chloramphenicol use and childhood leukaemia in Shanghai. *Lancet* **2**: 934–937
Smith MA, McCaffrey RP and Karp JE (1996) The secondary leukemias: challenges and reseach directions. *J Natl Cancer Inst* **88**: 407–418
Stiller CA, Allen MB and Eatock EM (1995) Childhood cancer in Britain: the national registry of childhood tumours and incidence rates 1978–87. *Eur J Cancer* **31**A: 2028–2034
Stocker JT and Askin FB (eds) (1998) *Pathology of Solid Tumors in Children*. Chapman & Hall Medical: London

*© 2000 Cancer Research Campaign*

Suk WA and Collman GW (1998) Genes and environment: their impact on children's health. *Environ Hlth Perspect* **106**: 817–820
Swanson J (1995) Magnetic fields from transmission lines: comparison of calculations and measurements. *IEE Proc-Gener Transm Distrib* **142**: No. 5
Swanson J and Kaune W (1999) Comparison of residential power-frequency magnetic fields away from appliances in different countries. *Bioelectromagnetics* **20**: 244–254
Taylor GM, Dearden S, Payne N, Ayres M, Gokhale DA, Birch JM, Blair V, Stevens RF, Will AM and Eden OB (1998) Evidence that an *HLA-DQA1-DQB1* haplotype influences susceptibility to childhood common acute lymphoblastic leukaemia in boys provides further support for an infection-related aetiology. *Br J Cancer* **78**: 561–565
Townsend P, Phillimore P and Beattie A (1988) *Health and Deprivation: Inequality and the North*. Croom Helm: London
United Nations Scientific Committee on the Effects of Atomic Radiation (1993) *Sources and effects of Jonizing Radiation*. *1993 Report to the General Assembly*. Appendix F, pp. 619–728. United Nations: New York
van Dongen JJM and Adriaansen HJ (1996) Immunobiology of leukemia. In: *Leukemia*, Henderson ES, Lister TA and Greaves MF (eds), pp. 83–130. WB Saunders: Philadelphia
van Steensel-Moll HA, Valkenburgh HA and Van Zanen GE (1985) Childhood leukemia and parental occupation: a register-based case–control study. *Am J Epidemiol* **121**: 216–224
Wertheimer N and Leeper E (1979) Electrical wiring configurations and childhood cancer. *Am J Epidemiol* **109**: 273–284
WHO (1990) *International Classification of Diseases for Oncology*, 2nd Edn, Percy C, Van Holten V and Muir C (eds). World Health Organization: Geneva
WHO (1992) *International Statistical Classification of Diseases and Related Health Problems*, Tenth Revision. World Health Organization: Geneva
Wrixon AD, Green BMR, Lomas PR, Miles JCH, Cliff KD, Francis EA, Driscoll CMH, James AC and O'Riordan MC (1988) *Natural Radiation Exposure in UK Dwellings*. NRPB-R190. National Radiological Protection Board, Chilton

 *© 2000 Cancer Research Campaign*
